# Inhibitory Effects of Coumarin Derivatives on Tyrosinase

**DOI:** 10.3390/molecules26082346

**Published:** 2021-04-17

**Authors:** Eon-Joo Roh

**Affiliations:** Interdisciplinary Program in Biotechnology, Graduate School, Changwon National University, Changwon 51140, Korea; no670@changwon.ac.kr or medlife99@gmail.com

**Keywords:** geranyloxycoumarin, tyrosinase, coumarin, inhibitor, melanin

## Abstract

In this study, a series of coumarin derivatives were synthesized and their inhibitory effects on the activity of mushroom tyrosinase were evaluated. As a result of measuring the inhibition of tyrosinase activity of these derivatives, the compounds **3e** (1.05 μM), **3f** (0.83 μM), **3h** (0.85 μM), **3i** (1.05 μM), and **3k** (0.67 μM) of the geranyloxycoumarin derivatives were highly active at a concentration of 0.8%. The geranyloxycoumarin derivatives exhibited better activity than the hydroxycoumarin derivatives. Among the geranyloxycoumarin derivatives, compound **3k** was two times more active than arbutin, a positive control, at a concentration of 0.4%. The above results suggest that geranyloxycoumarin derivatives have great potential for application as functional cosmetic ingredients with tyrosinase-inhibiting activity.

## 1. Introduction

Tyrosinase is known to be a multifunctional copper-containing enzyme from the oxidase superfamily, and it is the key protein involved in the biosynthesis of the large biological pigment, melanin [1]. Melanin is produced by melanocytes in the basal layer of the epidermis [2]. Tyrosinase promotes hydroxylation of L-Tyrosine to L-DOPA (L-3,4-dihydroxyphenylalanine) in the process of melanin biosynthesis, then promotes oxidation from L-DOPA to dopaquinone. Subsequently, dopaquinone forms melanin through several stages [3]. Melanin plays an important role in human skin, but excessive melanin formation caused by abnormal melanin production can cause pigmentation disorders, such as freckles, blemishes, and age spots [4]. These excessive melanin expressions can be suppressed by effective tyrosinase inhibitors. 

Tyrosinase inhibitors include hydroquinone [5], arbutin [6], kojic acid [7], ascorbic acid [8], and ellagic acid [9]. Hydroquinone is potentially mutagenic in mammalian cells and is associated with several side effects, including contact dermatitis [10]. Arbutin is chemically unstable in its natural form and potentially toxic to the bone marrow [6]. Kojic acid is restricted to use in cosmetics [11] due to carcinogenicity and instability during storage. Ellagic acid is insoluble and has low bioavailability [9]. Ascorbic acid is heat-sensitive and easily decomposed [12]. Because of these problems, tyrosinase inhibitors previously used have limited use, or are only permitted to be used at low concentrations.

Coumarin (2H-chromen-2-one, benzo-α-pyrone) and its derivatives are widely distributed in plants and are especially abundant in the bark and leaves of plants [13,14]. In addition, coumarin is a fragrant chemical compound of benzopyrone, notably found in cinnamon extracts [15,16,17]. Coumarins have shown evidence of many biological activities. The biomedical activities of coumarin include anti-HIV [18], anti-inflammatory [19], anti-cancer [20], anti-diabetes [21], and whitening effects [22]. Despite the many pharmaceutical effects of coumarin, the skin-protective function of coumarin has not been actively studied. In this study, the discovery of whitening active ingredients is urgent, and, in order to prepare for demand, this study aimed to find raw materials to be used in functional cosmetics by synthesizing derivatives of coumarin known to have whitening effects as lead materials.

## 2. Results and Discussion

### 2.1. Synthesis

**Figure 1:**
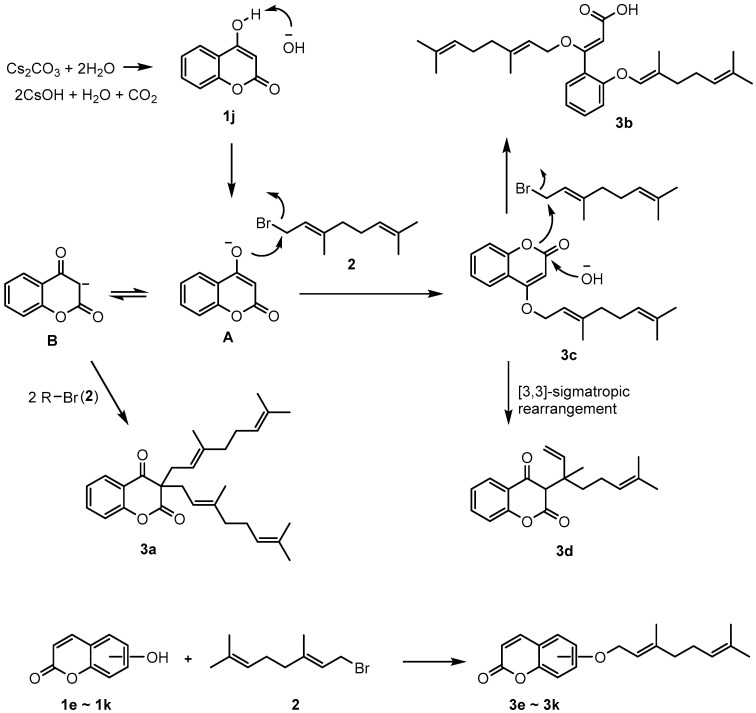
Reaction between hydroxycoumarin and alkenyl halide.

When there is an OH group at the 4-position of coumarin, geranyloxycoumarin derivatives are C-alkylated, with 18% O-alkylated coumarin **3c**, when reacting with geranyl bromide. This is due to the base-induced keto-enol tautomerization. As shown in Figure 1, **3a** was reacted with two equivalents of geranyl bromide (**2**) and keto form B to obtain an 11% yield. On the other hand, it is reported that **3d** [23] is formed by the rearrangement of O-alkylated coumarin (**3c**). It was confirmed that the formation of **3d** was generated through [3,3]-sigma tropic rearrangement by refluxing the isolated **3c** under acetonitrile solvent. As a result of these, the reaction between **1j** and excess geranyl bromide (**2**) gave the products **3a**, **3b**, and **3d**. Additionally, in this reaction, the yield of 4-geranyloxycoumarin (**3c**) obtained using triethylamine (TEA) as a base was as low as 2%, and **3a**, **3b**, and **3d** were not formed. However, under basic conditions, with K_2_CO_3_, CsOH, Cs_2_CO_3_, and Ag_2_CO_3_ as bases, geranyloxycoumarin derivative **3b** was obtained in a 35% yield; it had an open coumarin ring structure due to moisture in the atmosphere (Figure 1).

Except the coumarin with an OH group at the 4-position, which follows the reaction scheme shown in Figure 1, coumarins with OH groups at 3-, 5-, 6-, 7-, and 8-positions gave good yields of geranyloxycoumarin derivatives (**3e**–**3l**) when reacted with geranyl bromide under weakly basic conditions. As a result of the reaction between hydroxycoumarin (e.g., 6-hydroxycoumarin) and geranyl bromide in the presence of cesium carbonate and acetonitrile at room temperature, a general O-alkylated compound could be obtained in good yield. As shown in Figure 1, new coumarin derivatives were obtained by reacting various hydroxycoumarins and geranyl bromide under the given conditions. 

The geranyloxycoumarin derivatives were synthesized by reacting a hydroxycoumarin with geranyl bromide in acetonitrile solvent at room temperature for 3 h in the presence of various bases (NaOH, K_2_CO_3_, triethylamine (TEA), CsOH, Cs_2_CO_3_, or Ag_2_CO_3_). Geranyloxy derivatives (**3e–3l**) were obtained in good yield.

### 2.2. Tyrosinase Inhibitory Activity 

Tyrosinase is an enzyme that plays the most important role in the production of melanin from tyrosine when the skin is exposed to UV rays. Melanin is produced by enzymes, such as tyrosinase-related protein-1 (TRP-1) and dopachrome tautomerase (TRP-2), using tyrosine as a substrate from melanosomes in the melanocytes in the basal layer [23]. Melanin is produced by TRP-1 and TRP-2 as eumelanin, which appears black and brown, and pheomelanin, which appears yellow and red. Tyrosinase, a copper-containing metalloenzyme, is found in epidermal melanocytes, as well as the pigment epithelia of the retina, iris, and ciliary body of the eye [24]. Tyrosinase is one of the enzymes responsible for skin pigmentation in mammals. Melanin production can be inhibited by inhibiting the tyrosinase activity, thereby preventing the induction of melasma, freckles, and senile erythema [25].

**Figure 2:**
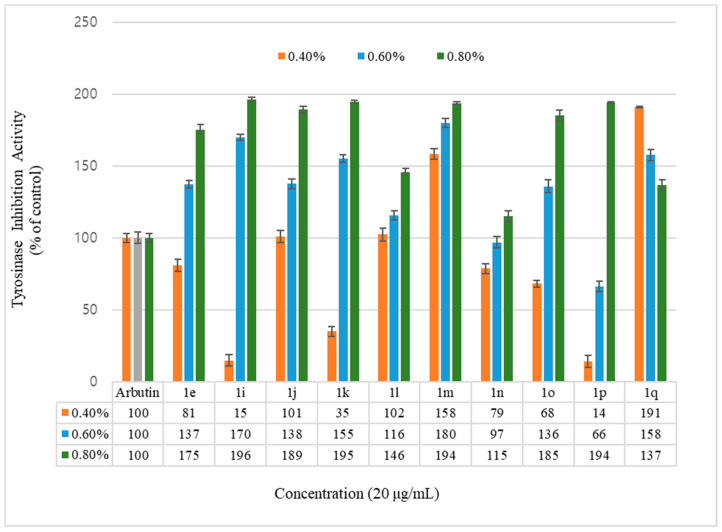
Mushroom tyrosinase activity effects of hydroxycoumarin derivatives and reference. Results are expressed as means ± SEMs.

**Figure 3:**
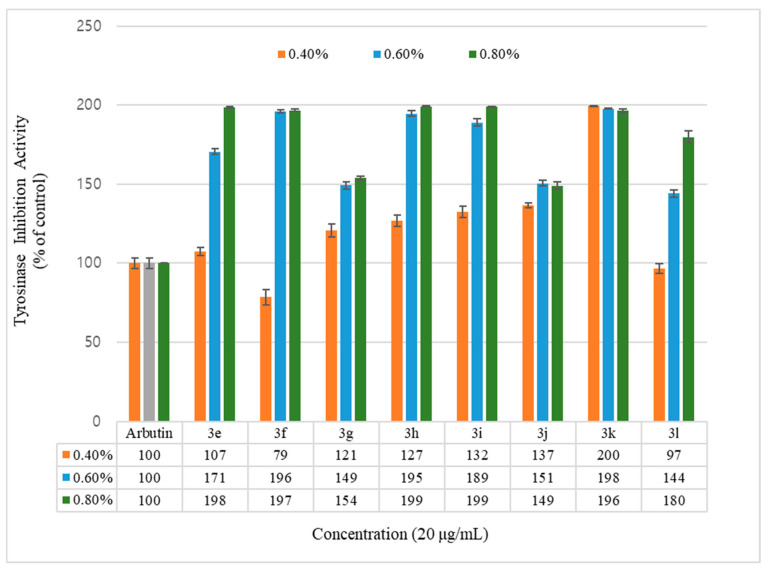
Mushroom tyrosinase activity effects of geranyloxycoumarin derivatives and reference. Results are expressed as means ± SEMs.

To evaluate the whitening effect of the coumarin derivatives, their ability to inhibit the tyrosinase activity was estimated. The coumarin derivatives were compared and evaluated by dividing them into the geranyloxycoumarin group (Table 1), containing the synthesized coumarin derivatives, and the hydroxycoumarin group (Table 2). After the compounds were treated at different concentrations of 0.4%, 0.6%, and 0.8%, the inhibitory ability of tyrosinase activity was calculated based on the following equation, and arbutin was used as a positive control. The results are shown in Figure 2 and Figure 3. 

Inhibition rate (%) = [(B − S)/B] × 100 

Here, B and S are the absorbances for the blank and samples. Arbutin was used as a reference standard inhibitor for comparison.

Inhibition of the tyrosinase activity was measured for the geranyloxycoumarin-derived compounds, **3e**–**3l**, and the hydroxycoumarin-derived compounds, **1e**–**1q**. The activity of the geranyloxy coumarin derivatives was found to be better than that of the hydroxycoumarin derivatives. The inhibitory activities of the eight geranyloxycoumarin derivatives at 0.4% concentration were similar to or better than that of arbutin, with the exception of compound **3f**, and markedly superior at 0.6% and 0.8% concentrations. In particular, compounds **3e**, **3f**, **3h**, **3i**, and **3k** among the geranyloxycoumarin derivatives, and compound **1i** among the hydroxycoumarin derivatives, were 1.9 times more active than arbutin at 0.8% concentration, and compound **3k** was two times more active than arbutin at a low concentration of 0.4%. The inhibitory effect of tyrosinase increased in a concentration-dependent manner, but the activities of compounds **3k** and **1q** were the highest at the lowest concentration of 0.4%. The activity tended to decrease with increasing concentration. The reason for the high tyrosinase-inhibitory activity of **3k** is presumed to have played an important role due to the interaction between the two unsaturated geranyloxy groups bonded to the R4 and R5 positions and the tyrosinase. However, it is estimated that the decrease in tyrosinase-inhibitory activity of 6-hydroxycoumarin (**1q**) is because the 6-OH group of the coumarin skeleton interferes with the interaction between tyrosinase as the concentration increases. 

## 3. Materials and Methods

### 3.1. Materials

In this study, geranyloxycoumarin derivatives were synthesized using substituted hydroxycoumarin (procured from TCI and AlfaAesar, Tokyo, Japan). Geranyl bromide (95%, Aldrich, St. Louis, MO, USA) was used as the alkenyl chain, and NaOH, triethylamine (Et3N), K_2_CO_3_, Cs_2_CO_3_, Ag_2_CO_3_, etc., were used as bases. Acetonitrile, ethyl acetate, dichloromethane, n-hexane, acetone, and ethanol were used as solvents. A nuclear magnetic resonance spectrometer (NMR spectrometer; BRUKER AVANCE 400 MHz, BRUKER, Karlsruhe, Germany) was used for analysis. CDCl_3_ and CD_3_OD containing tetramethylsilane (TMS), which is an internal standard, were used as analytical solvents. Infrared spectroscopy was performed on an FT/IR-4200 (JASCO, Tokyo, Japan) spectrophotometer, and KBr pellets were prepared to confirm the functional groups in the compound (see Supplementary Materials). In addition, the melting point was measured without calibrating the temperature. A thermometer was mounted under a paraffin oil container, and the open glass capillary method was used.

### 3.2. Synthesis

General method for the synthesis of geranyloxycoumarin derivative: 7-hydroxycoumarin (1.0 mmol) was added to a reaction mixture containing 25 mL of acetonitrile, cesium carbonate (1.1 mmol), and geranyl bromide (1.2 mmol, 95%) in a 50 mL double-neck round-bottom flask equipped with a thermometer, condenser, magnetic bar, and stirrer. The reaction mixture was stirred at room temperature for 3 h. After confirming the consumption of the starting material by thin-layer chromatography following 3 h of mixing, the reaction product was cooled at room temperature and filtered through a glass filter. After filtration, the solvent was removed using a rotary evaporator. Dichloromethane (20 mL) was added to the reaction mixture, and the mixture was stirred for 5 min. Following this, it was filtered and washed twice with 10 mL dichloromethane. The filtrate was concentrated using a rotary evaporator, and the residue was subjected to silica gel column chromatography using a mixed solvent of hexane/dichloromethane (1:1 volume ratio) to obtain geranyloxycoumarin derivatives. The analysis results of the synthesized coumarin derivatives are below.

*3,3-bis((E)-3,7-dimethylocta-2,6-dien-1-yl)-chromane-2,4-dione (**3a**).* Colorless liquid, Yield: 11%; IR (KBr, cm^−1^): ν 2966 (C-H), 2917 (C-H), 2854 (C-H), 1772 (C=O), 1689 (C=O), 1611, 1461, 1288 (C-O), 1142, 755; ^1^H-NMR (400 MHz, CDCl_3_): δ 1.47 (s, 6H, 2CH_3_), 1.55 (s, 6H, 2CH_3_), 1.59 (s, 6H, 2CH_3_), 1.72–1.84 (m, 8H, 2(-CH_2_CH_2_-)), 2.70–2.80 (m, 2H, -CH_2_-), 2.82–2.89 (m, 2H, -CH_2_-), 4.86–4.95 (m, 4H, 4(=CH)), 7.14–7.17 (m, 1H, H-6), 7.21–7.25 (m, 1H, H-5), 7.58–7.63 (m, 1H, H-8), 7.89–7.93 (m, 1H, H-7); ^13^C-NMR (100 MHz, CDCl_3_): δ 16.21 (CH_3_), 17.52 (CH_3_), 25.49 (CH_3_), 26.33 (CH), 37.62 (CH), 39.65 (CH), 62.24 (C), 116.89 (CH), 117.49 (C), 119.53 (CH), 123.78 (CH), 124.66 (CH), 126.55 (CH), 131.32 (C), 136.82 (C), 140.59 (C), 154.89 (C), 170.56 (C), 194.66 (C) ppm; MS *m*/*z* = 434 (M+); Anal. Calcd for C_29_H_38_O_3_: C, 80.14; H, 8.81, Found: C, 80.09; H, 8.76.

*(E)-3-(((E)-3,7-dimethylocta-2,6-dien-1-yl)oxy)-3-(2-(((E)-3,7-dimethylocta-2,6-dien-1-yl)oxy)phenyl) acrylic acid (**3b**).* Colorless liquid, Yield: 35%; IR (KBr, cm^−1^): ν 3328 (bs OH), 3012 (Aromatic C-H), 2994 (aliphatic C-H), 1712 (C=O), 1112 (C-O), 928, 806; ^1^H-NMR (400 MHz, CDCl_3_): δ 1.54 (s, 6H, 2CH_3_), 1.58 (s, 6H, 2CH_3_), 1.62 (s, 6H, 2CH_3_), 1.85–2.01 (m, 8H, 2(-CH_2_CH_2_-)), 2.25–2.32 (m, 2H, -CH_2_-), 2.41–2.48 (m, 2H, -CH_2_-), 3.47–3.54 (m, 1H, =CH), 4.98–5.03 (m, 2H, -OCH_2_-), 5.06–5.11 (m, 2H, -OCH_2_-), 6.86–6.90 (m, 1H, H-3), 6.95–6.98 (m, 1H, H-5), 7.42–7.46 (m, 1H, H-4), 7.77 (dd, J = 1.8 Hz, 8.2Hz, 1H, H-2), 12.66 (s, OH, D_2_O exch.); ^13^C-NMR (100 MHz, CDCl_3_): δ 16.10 (CH_3_), 17.67 (CH_3_), 25.65 (CH_3_), 26.54 (CH_2_), 30.60 (CH_2_), 39.74 (CH_2_), 46.39(CH_2_), 118.56 (C), 118.68(CH), 119.65 (C), 121.10(CH), 124.11 (CH), 130.24 (C), 131.45 (C), 136.20 (C), 137.59 (C), 162.93 (C), 210.28 (C) ppm; MS *m*/*z* = 452 (M+); Anal. Calcd for C_29_H_40_O_4_: C, 76.95; H, 8.91, Found: C, 76.93; H, 8.91.

*(E)-4-(3,7-dimethylocta-2,6-dienyloxy)-2H-chromen-2-one (**3c**).* White powder, Yield: 18%, m.p. 47–48 °C; IR (KBr, cm^−1^): ν 2923 (C-H), 1718 (C=O), 1620, 1371, 1235 (C-O), 1182 (C-O), 1104 (C-O), 923, 817, 764, 751, 500; ^1^H-NMR (400 MHz, CDCl_3_): δ 1.62 (s, 3H, CH_3_), 1.69 (s, 3H, CH_3_), 1.77 (s, 3H, CH_3_), 2.08–2.18 (m, 4H, -CH_2_CH_2_-), 4.71 (d, *J* = 6.7 Hz, 2H, -CH_2_-), 5.08–5.12 (m, 1H, =CH), 5.49–5.53 (m, 1H, =CH), 5.69 (s, 1H, H-3), 7.25–7.33 (m, H6 and H-8), 7.53–7.57 (m, 1H, H-7), 7.84 (dd, J = 2.2Hz, 5.8Hz, 1H, H-5); ^13^C-NMR (100 MHz, CDCl_3_): δ 16.82 (CH_3_), 17.75 (CH_3_), 25.70 (CH_3_), 26.19 (CH), 39.50 (CH), 66.27 (CH), 90.64 (CH), 115.92 (C), 116.75 (CH), 117.06 (CH), 123.19 (CH), 123.46 (CH), 123.83 (CH), 132.13 (CH), 132.31 (C), 143.74 (C), 153.35 (C), 163.15 (C), 165.61 (C) ppm; MS *m*/*z* = 298 (M+); Anal. Calcd for C_19_H_22_O_3_: C, 76.48; H, 7.43, Found: C, 76.49; H, 7.40.

*(E)-3-((3,7-dimethylocta-2,6-dien-1-yl)oxy)-2H-chromen-2-one (**3e**).* White solid, Yield: 87%; m.p. 72–73 °C; IR (KBr, cm^−1^): ν 3086 (Aromatic C-H), 3052 (Aromatic C-H), 2975 (Aliphatic C-H), 2917 (Aliphatic C-H), 2885 (Aliphatic C-H), 1745 (Carbonyl (ester -C=O)), 1638 (C=C bond), 1585, 1503, 1468, 1446, 1413, 1390, 1331, 1319, 1225, 1218, 1164, 1122, 996, 950, 938, 900, 864, 790, 761, 603; ^1^H-NMR (400 MHz, CDCl_3_): δ 1.57 (s, 3H, CH_3_), 1.62 (s, 3H, CH_3_), 1.73 (s, 3H, CH_3_), 2.03–2.13 (m, 4H, -CH_2_CH_2_-), 4.61 (d, *J* = 8.0 Hz, 2H, -CH_2_-), 5.02–5.06 (m, 1H, =CH), 5.46–5.51 (m, 1H, =CH), 6.79 (s, 1H, H-4), 7.20–7.28 (m, 2H, H6 and H-8), 7.32–7.37 (m, 2H, H-5 and H-7); ^13^C-NMR (100 MHz, CDCl3): δ 16.82 (CH_3_), 17.72 (CH_3_), 25.66 (CH_3_), 26.17 (CH), 39.50 (CH), 66.24 (CH), 113.69 (CH), 116.29 (CH), 117.96 (C), 119.82(CH), 123.59 (C), 124.64(CH), 126.37 (CH), 128.33 (CH), 132.00 (CH), 142.51 (C), 143.69 (C), 149.53 (C), 157.77 (C) ppm; MS *m*/*z* = 298 (M+); Anal. Calcd for C_19_H_22_O_3_: C, 76.48; H, 7.43, Found: C, 76.44; H, 7.42.

*(E)-7-((3,7-dimethylocta-2,6-dien-1-yl)oxy)-3-phenyl-2H-chromen-2-one (**3f**).* White solid, Yield: 92%; m.p. 104–105 °C; IR (KBr, cm^−1^): ν 3054 (Aromatic C-H), 3036 (Aromatic C-H), 2965 (Aliphatic C-H), 2909 (Aliphatic C-H), 2851 (Aliphatic C-H), 1707 (Carbonyl (ester -C=O)), 1606 (C=C bond), 1503, 1450, 1443, 1429, 1365, 1272, 11220, 1178, 1123, 1105, 1012, 990, 941, 827, 784, 691, 630; ^1^H-NMR(400 MHz, CDCl_3_): ^1^H-NMR (400 MHz, CDCl_3_): δ 1.59 (s, 3H, CH_3_), 1.66 (s, 3H, CH_3_), 1.75 (s, 3H, CH_3_), 2.05–2.15 (m, 4H, -CH_2_CH_2_-), 4.60 (d, *J* = 8.0 Hz, 2H, -CH_2_-), 5.05–5.09 (m, 1H, =CH), 5.44–5.48 (m, 1H, =CH), 6.84–6.87 (m, 2H, H-6 and H-8), 7.34–7.44 (m, 4H), 7.65–7.68 (m, 2H), 7.74 (s, 1H, H-4); ^13^C-NMR (100 MHz, CDCl_3_): δ 16.76 (CH_3_), 17.69 (CH_3_), 25.64 (CH_3_), 26.20 (CH_2_), 39.48 (CH_2_), 65.47 (CH_2_), 101.12 (CH), 113.19 (CH), 113.39 (CH), 118.38 (CH), 123.58 (C), 124.63 (CH), 128.36 (CH), 128.39 (CH), 128.74 (CH), 131.94 (C), 135.03 (C), 140.06 (C), 142.34 (CH), 155.23 (C), 160.95 (C), 161.88 (C) ppm; MS *m*/*z* = 374 (M+); Anal. Calcd for C_25_H_26_O_3_: C, 80.18; H, 7.00, Found: C, 80.16; H, 6.99.

*(E)-7-((3,7-dimethylocta-2,6-dien-1-yl)oxy)-4-methyl-2H-chromen-2-one (**3g**).* White solid, Yield: 91%; m.p. 54–55 °C; IR(KBr, cm^−1^): ν 3078 (Aromatic C-H), 3028 (Aromatic C-H), 2964 (Aliphatic C-H), 2917 (Aliphatic C-H), 2856 (Aliphatic C-H), 1726 (Carbonyl (ester -C=O)), 1617 (C=C bond), 1508, 1441, 1420,1390, 1345, 1278, 1257, 1199, 1154, 1134, 1070, 992, 982, 843, 825; ^1^H-NMR (400 MHz, CDCl_3_): δ 1.57 (s, 3H, CH_3_), 1.63 (s, 3H, CH_3_), 1.73 (s, 3H, CH_3_), 2.03–2.13 (m, 4H, -CH_2_CH_2_-), 2.37 (d, *J* = 1.1 Hz, 3H, CH_3_), 4.57 (d, *J* = 6.5 Hz, 2H, -CH_2_-), 5.03–5.07 (m, 1H, =CH), 5.42–5.46 (m, 1H, =CH), 6.10 (q, *J* = 1.2 Hz, 2.4 Hz, 1H, H-3), 6.79 (d, *J* = 2.5 Hz, 8.8 Hz, 1H, H-8), 6.84 (d, *J* = 2.5 Hz, 1H, H-6), 7.46 (dd, *J* = 2.6 Hz, 8.8 Hz, 1H, H-5); ^13^C-NMR (100 MHz, CDCl_3_): δ 16.79 (CH_3_), 17.73 (CH_3_), 18.70 (CH_3_), 25.68 (CH_3_), 26.24 (CH_2_), 39.53 (CH_2_), 65.44 (CH_2_), 101.59 (CH), 111.85 (CH), 112.94 (C), 113.47 (CH), 118.45 (CH), 123.63 (CH), 125.46 (CH), 131.97 (C), 142.35 (C), 152.61 (C), 155.25 (C), 161.41 (C), 161.95 (C) ppm; MS *m*/*z* = 312 (M+); Anal. Calcd for C_20_H_24_O_3_: C, 76.89; H, 7.74, Found: C, 76.88; H, 7.72.

*(E)-7-((3,7-dimethylocta-2,6-dien-1-yl)oxy)-4-(trifluoromethyl)-2H-chromen-2-one (**3h**).* White solid, Yield: 90%; m.p. 62–63 °C; IR (KBr, cm^−1^): ν 3077 (Aromatic C-H), 3030 (Aromatic C-H), 2974 (Aliphatic C-H), 2914 (Aliphatic C-H), 2894 (Aliphatic C-H), 1730 (Carbonyl (ester -C=O)), 1609 (C=C bond), 1556, 1516, 1452, 1427, 1400, 1351, 1275, 1215, 1192, 1166, 1137, 1014, 999, 958, 872, 818, 781, 715, 649, 626; ^1^H-NMR (400 MHz, CDCl_3_): δ 1.57 (s, 3H, CH_3_), 1.62 (s, 3H, CH_3_), 1.73 (s, 3H, CH_3_), 2.03–2.13 (m, 4H, -CH_2_CH_2_-), 4.59 (d, *J* = 4.0 Hz, -OCH_2_-), 5.01–5.06 (m, 1H, =CH), 5.40–5.45 (m, 1H, =CH), 6.57 (s, 1H, H-3), 6.83 (d, *J* = 2.5 Hz, 1H, H-8), 6.88 (dd, *J* = 2.5 Hz, 9.0 Hz, 1H, H-6), 7.56–7.59 (m, 1H, H-5); ^13^C-NMR (100 MHz, CDCl_3_): δ 16.79 (CH_3_), 17.70 (CH_3_), 25.65 (CH_3_), 26.20 (CH_2_), 39.50 (CH_2_), 65.67 (CH_2_), 102.13 (CH), 106.88 (CH), 112.04 (q, ^3^*J*_CF_ = 5.7 Hz), 113.98 (C), 120.25(CH), 123.00 (CH), 123.54(CH), 126.24 (q, ^3^*J*_CF_ = 2.0 Hz, CH), 132.02 (C), 141.61 (q, ^2^*J*_CF_ = 32.7 Hz, CCF_3_), 142.78 (C), 156.31 (C), 159.49 (C), 162.86 (C) ppm; MS *m*/*z* = 366 (M+); Anal. Calcd for C_20_H_21_F_3_O_3_: C, 65.57; H, 5.78, Found: C, 65.54; H, 5.76.

*(E)-7-((3,7-dimethylocta-2,6-dien-1-yl)oxy)-2H-chromen-2-one (**3i**).* White solid, Yield: 94%; m.p. 68–69 °C; IR (KBr, cm^−1^): ν 3082 (Aromatic C-H), 3053 (Aromatic C-H), 2972 (Aliphatic C-H), 2896 (Aliphatic C-H), 2879 (Aliphatic C-H), 2849 (Aliphatic C-H), 2833 (Aliphatic C-H), 1728 (Carbonyl (ester -C=O)), 1611 (C=C bond), 1507, 1452, 1430, 1403, 1369, 1348, 1280, 1234, 1201, 1165, 1126, 1103, 1022, 990, 889, 852, 830, 776, 760; ^1^H-NMR (400 MHz, CDCl_3_): δ 1.60 (s, 3H, CH_3_), 1.66 (s, 3H, CH_3_), 1.75 (s, 3H, CH_3_), 2.06–2.15 (m, 4H, -CH_2_CH_2_-), 4.59 (d, *J* = 6.7 Hz, 2H, -CH_2_-), 5.05–5.09 (m, 1H, =CH), 5.44–5.48 (m, 1H, =CH), 6.24 (d, *J* = 9.5 Hz, 1H, H-3), 6.81 (d, *J* = 2.5 Hz, 1H, H-6), 6.84 (dd, *J* = 2.4 Hz, 8.4 Hz, 1H, H-8), 7.36 (d, *J* = 8.6 Hz, 1H, H-5), 7.63 (d, *J* = 9.5 Hz, 1H, H-4); ^13^C-NMR (100 MHz, CDCl_3_): δ 16.37 (CH_3_), 17.31 (CH_3_), 25.26 (CH_3_), 25.82 (CH_2_), 39.11 (CH_2_), 65.08 (CH_2_), 101.18 (CH), 112.01 (CH), 112.56 (C), 112.84 (CH), 117.99 (CH), 123.20 (CH), 128.26 (CH), 131.57 (C), 141.98 (C), 143.04 (CH), 155.47 (C), 160.90 (C), 161.74 (C) ppm; MS *m*/*z* = 298 (M+); Anal. Calcd for C_19_H_22_O_3_: C, 76.48; H, 7.43, Found: C, 76.45; H, 7.41.

*(E)-6-chloro-7-((3,7-dimethylocta-2,6-dien-1-yl)oxy)-4-methyl-2H-chromen-2-one (**3j**).* White solid, Yield: 90%; m.p. 95–96 °C; IR (KBr, cm^−1^): ν 3078 (Aromatic C-H), 3003 (Aromatic C-H), 2965 (Aliphatic C-H), 2916 (Aliphatic C-H), 2856 (Aliphatic C-H), 2854 (Aliphatic C-H), 1728 (Carbonyl (ester -C=O)), 1609 (C=C bond), 1494, 1414, 1388, 1378, 1320, 1274, 1205, 1157, 1083, 1047, 982, 883, 829; ^1^H-NMR (400 MHz, CDCl_3_): δ 1.59 (s, 3H, CH_3_), 1.65 (s, 3H, CH_3_), 1.77 (s, 3H, CH_3_), 2.06–2.15 (m, 4H, -CH_2_CH_2_-), 2.38 (d, *J* = 1.2 Hz, 3H, CH_3_), 4.69 (d, *J* = 6.4Hz, 2H, -CH_2_-), 5.04–5.08 (m, 1H, =CH), 5.44–5.48 (m, 1H, =CH), 6.16 (dd, *J* = 1.4 Hz, 2.6 Hz, 1H, H-3), 6.83 (s, 1H), 7.56 (s, 1H); ^13^C-NMR (100 MHz, CDCl_3_): δ 16.89 (CH_3_), 17.73 (CH_3_), 18.65 (CH_3_), 25.65 (CH_3_), 26.19 (CH_2_), 39.50 (CH_2_), 66.63 (CH_2_), 101.57 (CH), 112.74 (CH), 113.65 (C), 118.03 (CH), 119.34 (C), 123.54(CH), 125.32 (CH), 132.01 (C), 142.66 (C), 151.70 (C), 153.52 (C), 156.93 (C), 160.79 (C) ppm; MS *m*/*z* = 346 (M+); Anal. Calcd for C_20_H_23_ClO_3_: C, 69.26; H, 6.68, Found: C, 69.24; H, 6.65.

*6-(((E)-3,7-dimethylocta-2,6-dien-1-yl)oxy)-7-(((Z)-3,7-dimethylocta-2,6-dien-1-yl)oxy)-4-methyl-2H-chromen-2-one (**3k**).* White solid, Yield: 86%; m.p. 63–64 °C; IR (KBr, cm^−1^): ν 3065 (Aromatic C-H), 2937 (Aliphatic C-H), 2917 (Aliphatic C-H), 2854 (Aliphatic C-H), 1708 (Carbonyl (ester -C=O)), 1612 (C=C bond), 1562, 1520, 1430, 1384, 1280, 1231, 1164, 984, 822; ^1^H-NMR (400 MHz, CDCl_3_): δ 1.59 (s, 6H, 2CH_3_), 1.64 (s, 6H, 2CH_3_), 1.77 (s, 6H, 2CH_3_), 2.06–2.15 (m, 8H, 2(-CH_2_CH_2_-)), 2.37 (d, *J* = 1.2 Hz, 3H, CH_3_), 4.67 (d, *J* = 6.4 Hz, 4H, 2(-CH_2_-)), 5.04–5.08 (m, 2H, 2(=CH)), 5.44–5.48 (m, 2H, 2(=CH)), 6.15 (dd, *J* = 1.4 Hz, 2.64 Hz, 1H, H-3), 6.83 (s, 1H, H-5), 7.55 (s, 1H, H-8); ^13^C-NMR (100 MHz, CDCl_3_): δ 17.04 (CH_3_), 17.17 (CH_3_), 18.01 (CH_3_), 19.14 (CH_3_), 25.95 (CH_3_), 26.52 (CH_3_), 26.59 (CH_3_), 39.81 (CH_2_), 39.86 (CH_2_), 66.63 (CH_2_), 67.16 (CH_2_), 101.75 (CH), 108.76 (CH), 112.37 (C), 112.65 (CH), 119.06 (CH), 119.92 (C), 123.96 (CH), 132.22 (C), 141.50 (C), 141.91 (C), 145.76 (C), 149.76 (C), 152.70 (C), 153.07 (C), 161.95 (C) ppm; MS *m*/*z* = 346 (M+); Anal. Calcd for C_20_H_23_ClO_3_: C, 69.26; H, 6.68, Found: C, 69.24; H, 6.65.

*(E)-6-((3,7-dimethylocta-2,6-dien-1-yl)oxy)-4-methyl-2H-chromen-2-one (**3l**).* White solid, Yield: 89%; m.p. 57–58 °C; IR (KBr, cm^−1^): ν 3040 (Aromatic C-H), 2965 (aliphatic C-H), 2925 (aliphatic C-H), 2884 (aliphatic C-H), 1712(C=O), 1673, 1571, 1493, 1428, 1386, 1275, 1238 (C-O), 1167, 990, 926, 838; ^1^H-NMR (400 MHz, CDCl_3_): δ 1.60 (s, 3H, CH_3_), 1.67 (s, 3H, CH_3_), 1.77 (s, 3H, CH_3_), 2.06–2.17 (m, 4H, -CH_2_CH_2_-), 2.41 (d, *J* = 1.2 Hz, 3H, CH_3_), 4.59 (d, *J* = 6.6 Hz, 2H, -CH_2_-), 5.06–5.10 (m, 1H, =CH), 5.47–5.51 (m, 1H, =CH), 6.30 (q, *J* = 1.4 Hz, 2.6 Hz, 1H, H-3), 7.04 (d, *J* = 2.9 Hz, 1H, H-5), 7.13 (dd, *J* = 2.9 Hz, 9.0 Hz, 1H, H-7), 7.27 (d, *J* = 9.0 Hz, 1H, H-8); ^13^C-NMR (100 MHz, CDCl_3_): δ 16.74 (CH_3_), 17.72 (CH_3_), 18.74 (CH_3_), 25.68 (CH_3_), 26.27 (CH_2_), 39.55 (CH_2_), 65.56 (CH_2_), 108.82 (CH), 115.44 (CH), 117.89 (CH), 119.00 (CH), 119.32 (C), 120.43 (CH), 123.64 (CH), 131.96 (C), 141.96 (C), 147.84 (C), 152.02 (C), 155.20 (C), 161.03 (C) ppm; MS *m*/*z* = 312 (M+); Anal. Calcd for C_20_H_24_O_3_: C, 76.89; H, 7.74, Found: C, 76.86; H, 7.75.

### 3.3. Tyrosinase Inhibition Assay

During melanin synthesis, tyrosinase oxidizes tyrosine to DOPA, which is converted to DOPAquinone. The product of this reaction, DOPAquinone, has an absorbance maximum at 490 nm. The method for the oxidation of L-DOPA by tyrosinase, established by Fling et al. (1963), was followed [19]. Sodium phosphate buffer (50 mM, pH 7.2), 0.24 mM L-DOPA, inhibitor, and 53.3 units/mL mushroom tyrosinase (Sigma) were adjusted to 400 µL, and the change in absorbance at 490 nm was measured at room temperature for 10 min. The amount of DOPAchrome (ε = 3700 M^−1^·cm^−1^) produced was calculated based on the increase in enzyme absorbance. Arbutin was used as a positive control. All the chemicals and reagents were purchased from Sigma.

### 3.4. Statistical Analysis

The values are expressed as mean ± standard error (n = 3) and the biological significance *p* < 0.05 was determined by Student’s *t*-test.

## 4. Conclusions

In this study, geranyloxycoumarin derivatives were effectively synthesized using coumarin, which affects a number of physiological activities, as a lead compound. Various geranyloxycoumarin derivatives were obtained in high yields. Inhibition of the tyrosinase activity by various derivatives suggested that the geranyloxycoumarin derivatives exhibited better activity than the hydroxycoumarin derivatives. Among the geranyloxycoumarin derivatives, compound **3k** was two times more active than arbutin, a positive control, at a concentration of 0.4%.

The above results suggest that geranyloxycoumarin derivatives have great potential for application as functional cosmetic ingredients with tyrosinase-inhibiting activity.

## Figures and Tables

**Table 1:**
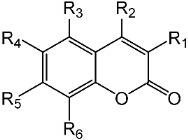
Synthesized geranyloxy coumarin compounds **3e**–**3l**.

Compound	R1	R2	R3	R4	R5	R6	Yield (%)
**3e**	Geranyloxy	H	H	H	H	H	87
**3f**	Ph	H	H	H	Geranyloxy	H	92
**3g**	H	CH_3_	H	H	Geranyloxy	H	91
**3h**	H	CF_3_	H	H	Geranyloxy	H	90
**3i**	H	H	H	H	Geranyloxy	H	94
**3j**	H	CH_3_	H	Cl	Geranyloxy	H	90
**3k**	H	CH_3_	H	Geranyloxy	Geranyloxy	H	86
**3l**	H	CH_3_	H	Geranyloxy	H	H	89

**Table 2:**
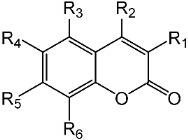
Hydroxycoumarin derivatives with functional groups in several positions **1e**–**1q**.

Compound	R1	R2	R	R4	R5	R6
**1e**	OH	H	H	H	H	H
**1i**	H	H	H	H	OH	H
**1j**	H	CH_3_	H	Cl	OH	H
**1k**	H	CH_3_	H	OH	OH	H
**1l**	H	CH_3_	H	OH	H	H
**1m**	CN	H	H	H	OH	H
**1n**	Cl	CH_3_	H	H	OH	H
**1o**	NO_2_	OH	H	H	H	H
**1p**	H	CH_2_COOH	H	H	OH	H
**1q**	H	H	H	OH	H	H

## Supplementary Materials

The following are available online.

## Sample Availability

Samples of the compounds are available from the authors.
