# Supplementary material for: Inhibitory Effects of Coumarin Derivatives on Tyrosinase
Source: Molecules. 2021 Apr 17;26(8):2346. doi: 10.3390/molecules26082346 (PMC8073051; doi:10.3390/molecules26082346)
Supplement: Supplementary file 1 [file molecules-26-02346-s001.zip › 3g-C NMR.pdf]

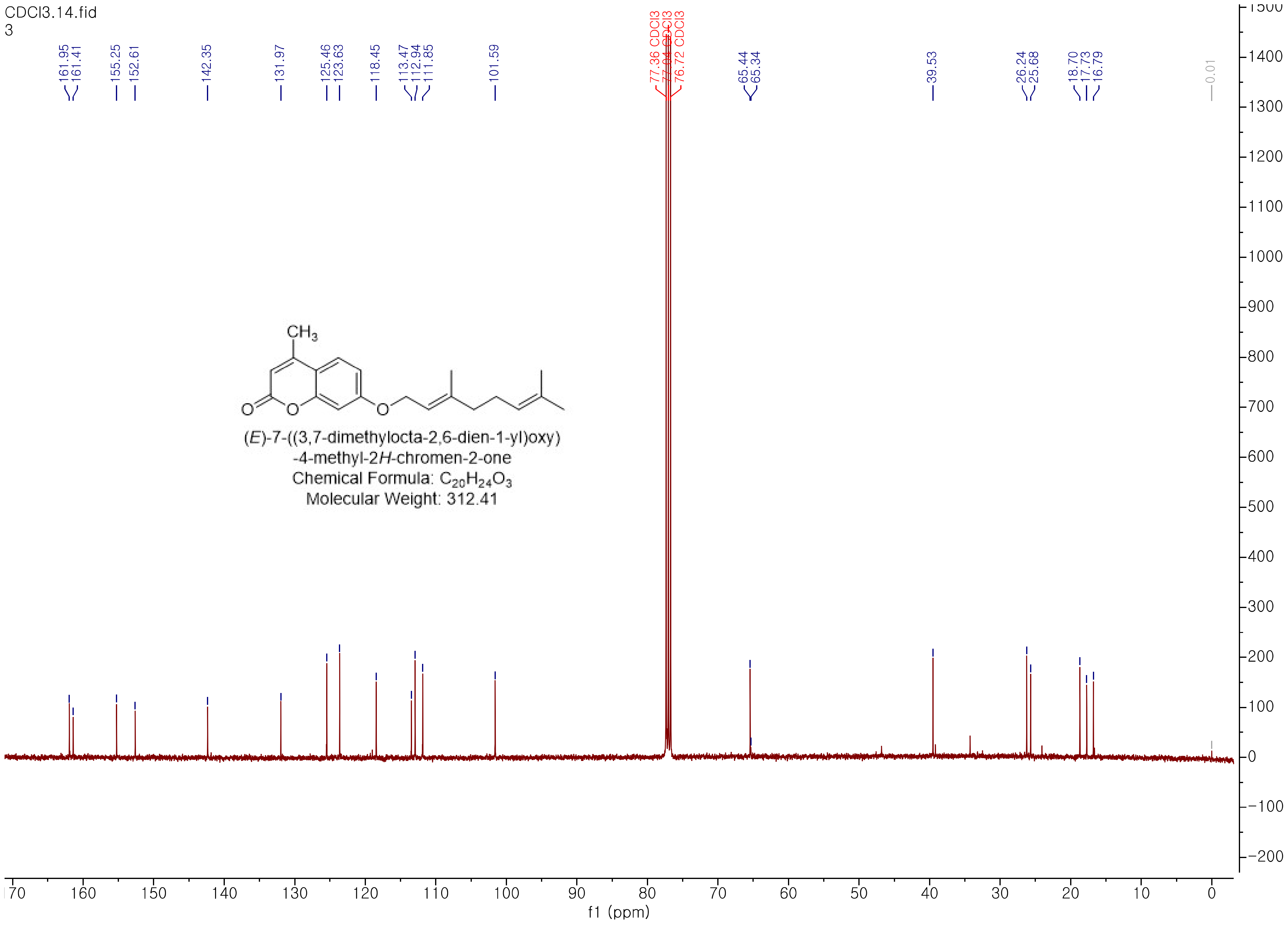

CDCl3.14.fid
3
161.95
161.41
155.25
152.61
142.35
131.97
125.46
123.63
118.45
113.47
112.94
111.85
101.59
77.36 CDCl3
77.04 CDCl3
76.72 CDCl3
65.44
65.34
39.53
26.24
25.68
18.70
17.73
16.79
0.01
CH3
O
O
O
(E)-7-((3,7-dimethylocta-2,6-dien-1-yl)oxy)
-4-methyl-2H-chromen-2-one
Chemical Formula: C20H24O3
Molecular Weight: 312.41
170
160
150
140
130
120
110
100
90
80
70
60
50
40
30
20
10
0
f1 (ppm)
1500
1400
1300
1200
1100
1000
900
800
700
600
500
400
300
200
100
0
−100
−200
