# Supplementary material for: Inhibitory Effects of Coumarin Derivatives on Tyrosinase
Source: Molecules. 2021 Apr 17;26(8):2346. doi: 10.3390/molecules26082346 (PMC8073051; doi:10.3390/molecules26082346)
Supplement: Supplementary file 1 [file molecules-26-02346-s001.zip › 3k-C NMR.pdf]

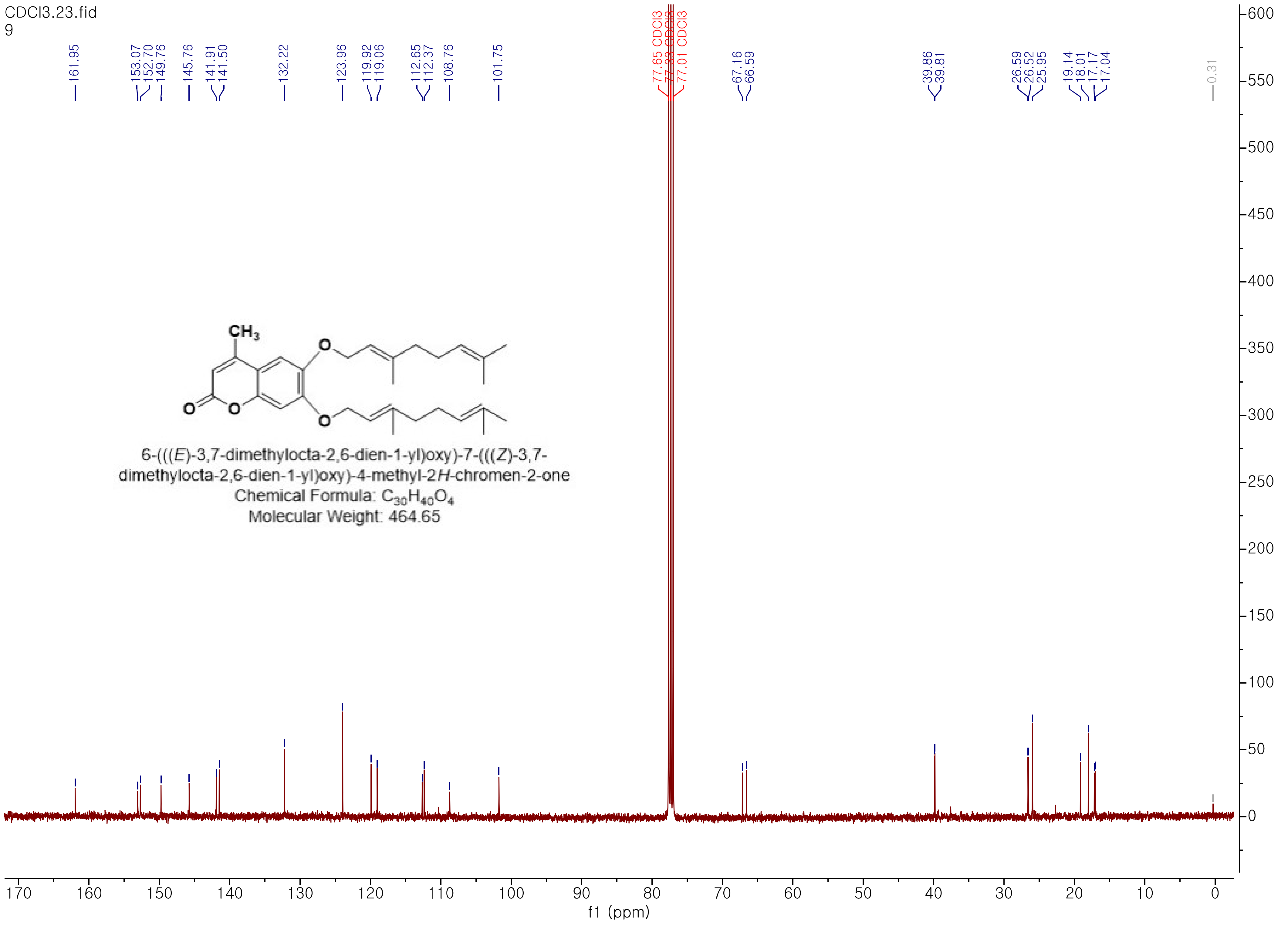
CDCl3.23.fid
9
161.95
153.07
152.70
149.76
145.76
141.91
141.50
132.22
123.96
119.92
119.06
112.65
112.37
108.76
101.75
77.65 CDCl3
77.33 CDCl3
77.01 CDCl3
67.16
66.59
39.86
39.81
26.59
26.52
25.95
19.14
18.01
17.17
17.04
0.31
CH3
O
O
O
O
6-(((E)-3,7-dimethylocta-2,6-dien-1-yl)oxy)-7-(((Z)-3,7-dimethylocta-2,6-dien-1-yl)oxy)-4-methyl-2H-chromen-2-one
Chemical Formula: C30H40O4
Molecular Weight: 464.65
600
550
500
450
400
350
300
250
200
150
100
50
0
170
160
150
140
130
120
110
100
90
80
70
60
50
40
30
20
10
0
f1 (ppm)
