# Supplementary material for: Inhibitory Effects of Coumarin Derivatives on Tyrosinase
Source: Molecules. 2021 Apr 17;26(8):2346. doi: 10.3390/molecules26082346 (PMC8073051; doi:10.3390/molecules26082346)
Supplement: Supplementary file 1 [file molecules-26-02346-s001.zip › ir-3e.pdf]

No.1

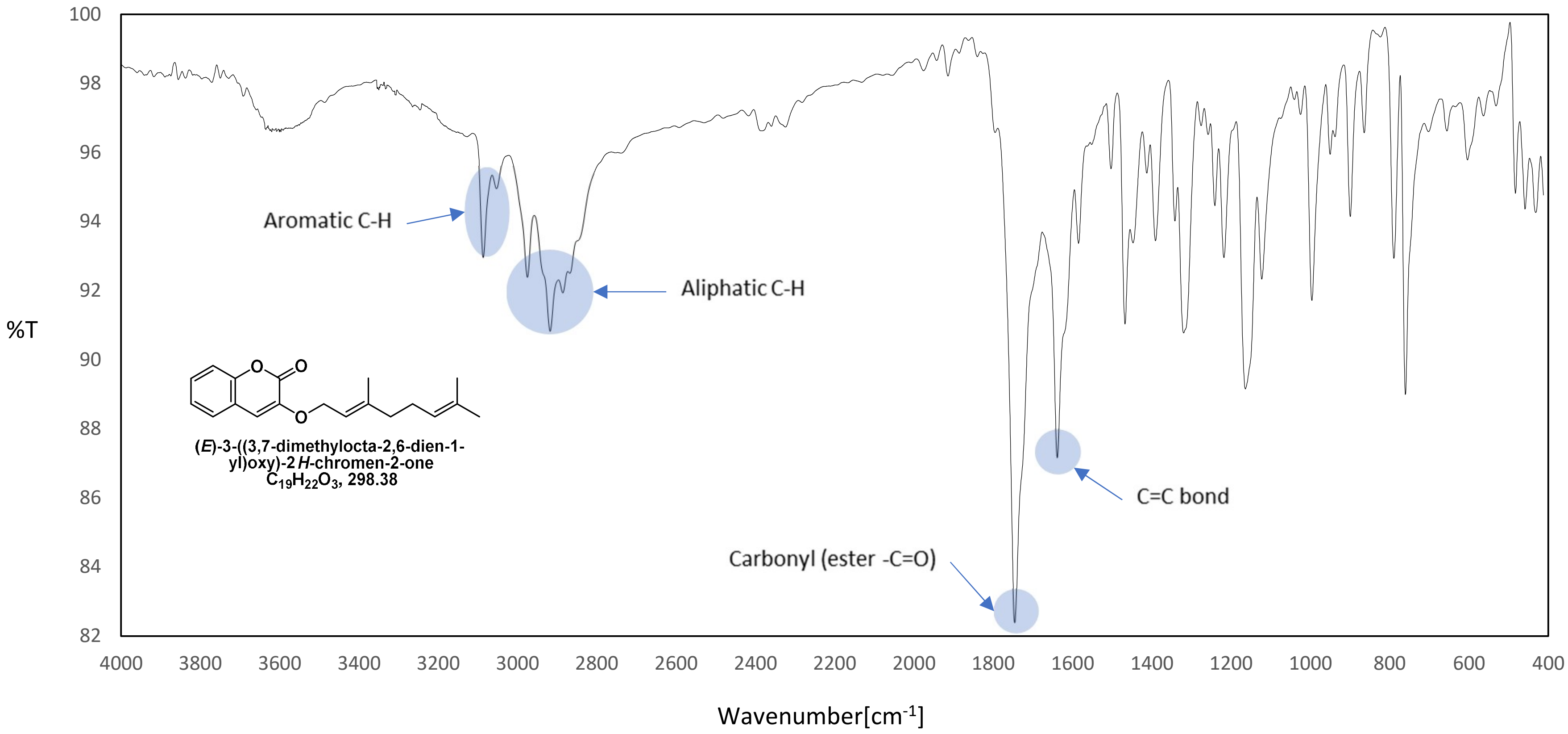

IR(KBr): 3086(Aromatic C-H), 3052(Aromatic C-H), 2975(Aliphatic C-H), 2917(Aliphatic C-H), 2885(Aliphatic C-H), 1745(Carbonyl (ester -C=O)), 1638(C=C bond), 1585, 1503, 1468, 1446, 1413, 1390, 1331, 1319, 1225, 1218, 1164, 1122, 996, 950, 938, 900, 864, 790, 761, 603 cm^{-1}
