# Supplementary material for: Inhibitory Effects of Coumarin Derivatives on Tyrosinase
Source: Molecules. 2021 Apr 17;26(8):2346. doi: 10.3390/molecules26082346 (PMC8073051; doi:10.3390/molecules26082346)
Supplement: Supplementary file 1 [file molecules-26-02346-s001.zip › ir-3f.pdf]

No.2

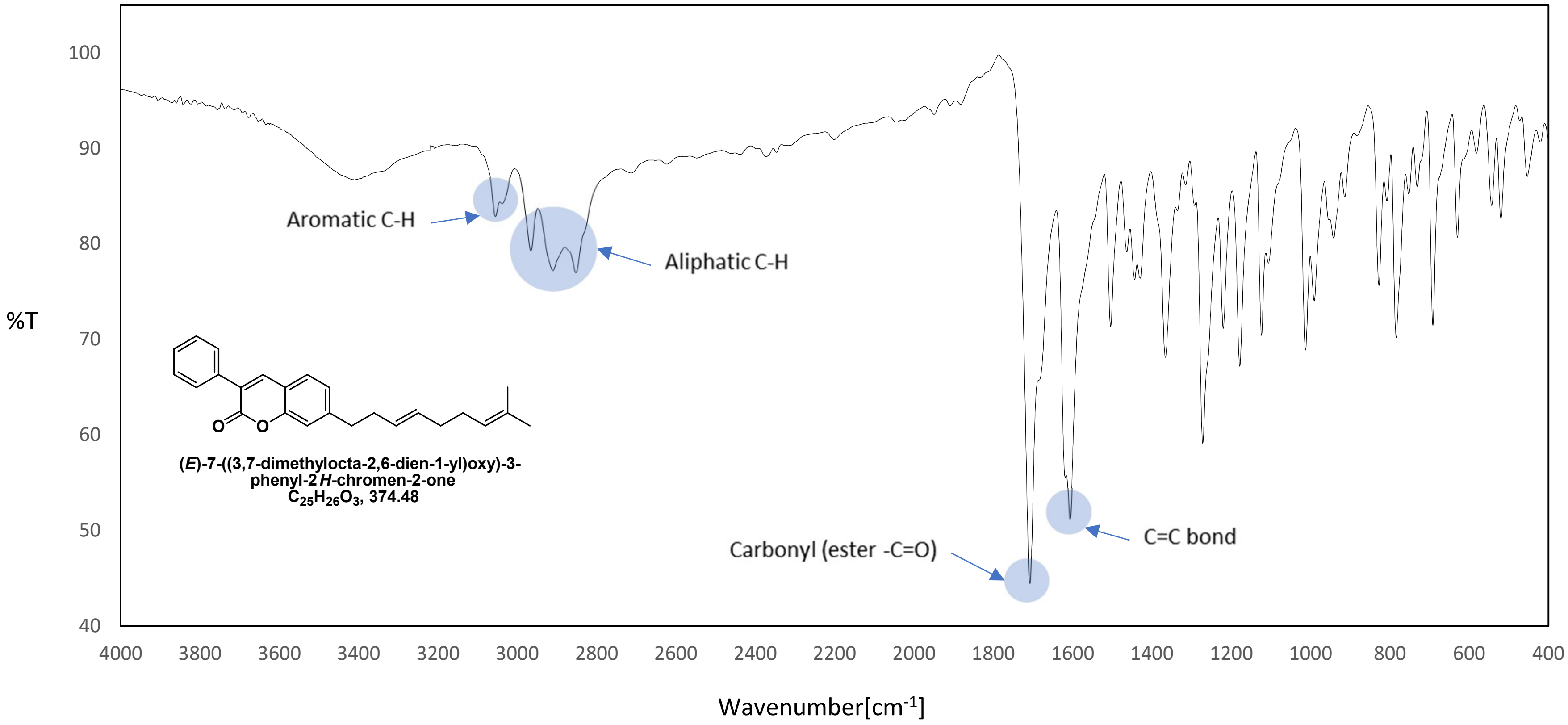

IR(KBr): 3054(Aromatic C-H), 3036(Aromatic C-H), 2965(Aliphatic C-H), 2909(Aliphatic C-H), 2851(Aliphatic C-H), 1707(Carbonyl (ester -C=O)), 1606(C=C bond), 1503, 1450, 1443, 1429, 1365, 1272, 11220, 1178, 1123, 1105, 1012, 990, 941, 827, 784, 691, 630 cm^{-1}
