# Supplementary material for: Inhibitory Effects of Coumarin Derivatives on Tyrosinase
Source: Molecules. 2021 Apr 17;26(8):2346. doi: 10.3390/molecules26082346 (PMC8073051; doi:10.3390/molecules26082346)
Supplement: Supplementary file 1 [file molecules-26-02346-s001.zip › ir-3h.pdf]

No.4

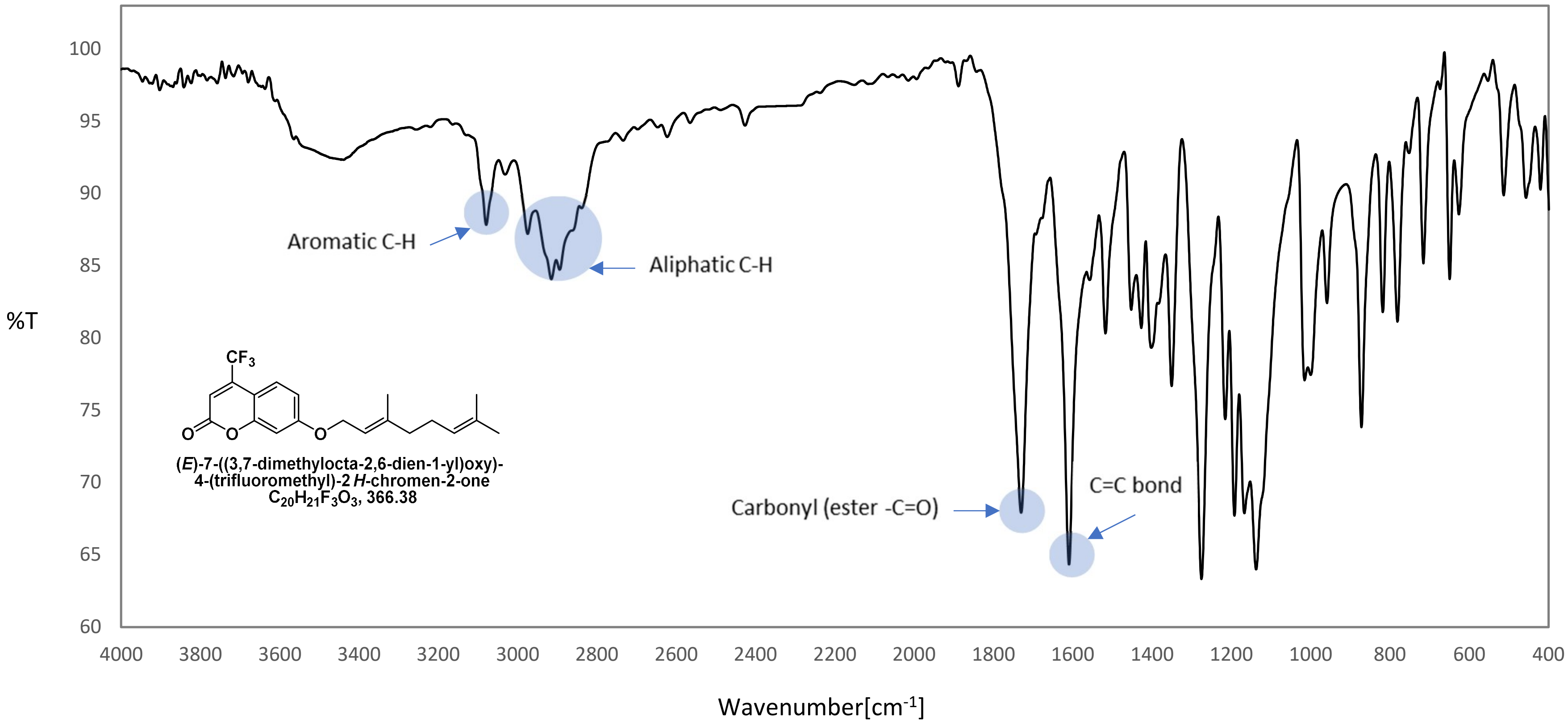

IR(KBr): 3077(Aromatic C-H), 3030(Aromatic C-H), 2974(Aliphatic C-H), 2914(Aliphatic C-H), 2894(Aliphatic C-H), 1730(Carbonyl (ester -C=O)), 1609(C=C bond), 1556, 1516, 1452, 1427, 1400, 1351, 1275, 1215, 1192, 1166, 1137, 1014, 999, 958, 872, 818, 781, 715, 649, 626 cm^{-1}
