# Supplementary material for: Inhibitory Effects of Coumarin Derivatives on Tyrosinase
Source: Molecules. 2021 Apr 17;26(8):2346. doi: 10.3390/molecules26082346 (PMC8073051; doi:10.3390/molecules26082346)
Supplement: Supplementary file 1 [file molecules-26-02346-s001.zip › ir-3i.pdf]

No.6

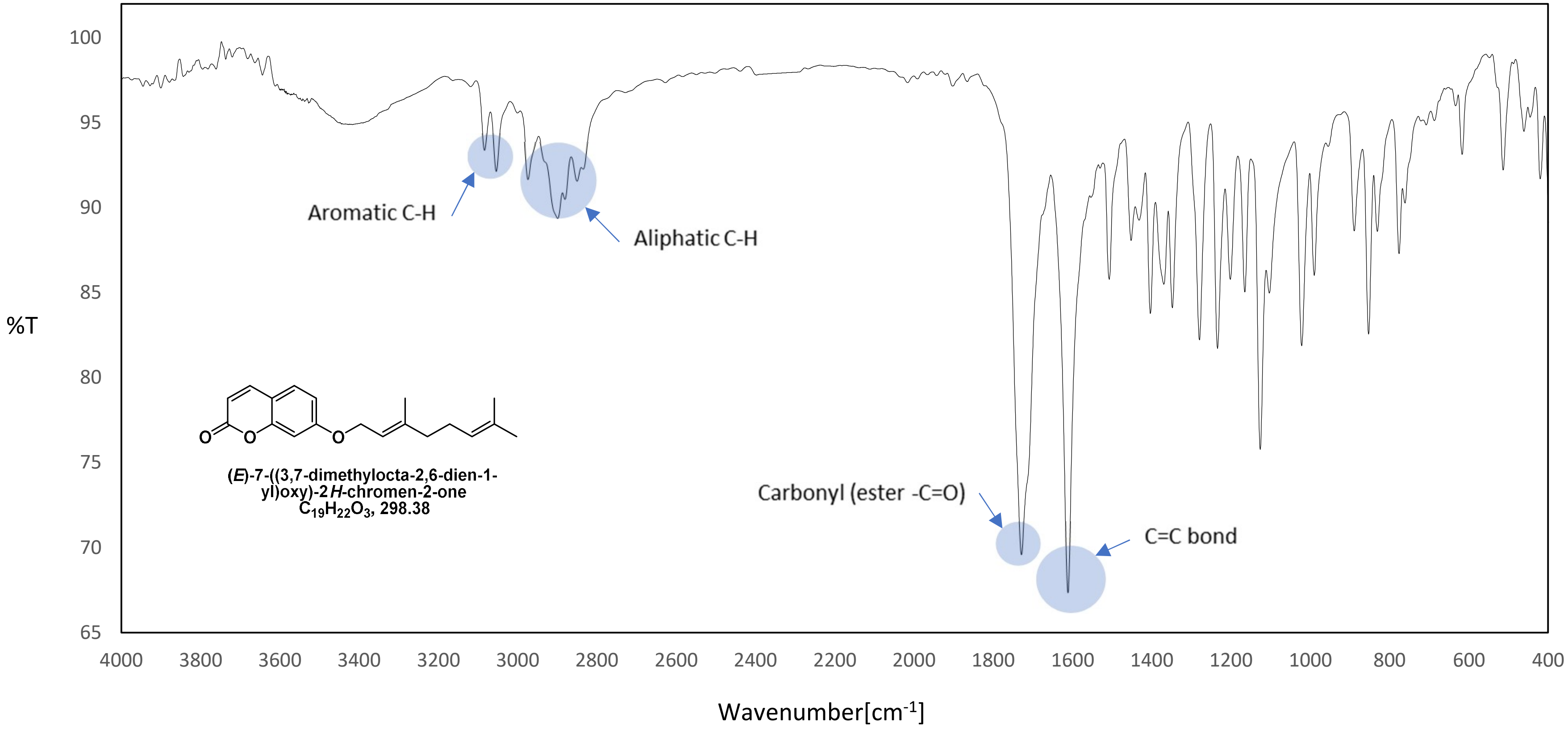

IR(KBr): 3082(Aromatic C-H), 3053(Aromatic C-H), 2972(Aliphatic C-H), 2896(Aliphatic C-H), 2879(Aliphatic C-H), 2849(Aliphatic C-H), 2833(Aliphatic C-H), 1728(Carbonyl (ester -C=O)), 1611(C=C bond), 1507, 1452, 1430, 1403, 1369, 1348, 1280, 1234, 1201, 1165, 1126, 1103, 1022, 990, 889, 852, 830, 776, 760 cm^{-1}
