# Supplementary material for: Inhibitory Effects of Coumarin Derivatives on Tyrosinase
Source: Molecules. 2021 Apr 17;26(8):2346. doi: 10.3390/molecules26082346 (PMC8073051; doi:10.3390/molecules26082346)
Supplement: Supplementary file 1 [file molecules-26-02346-s001.zip › ir-3j.pdf]

No.8

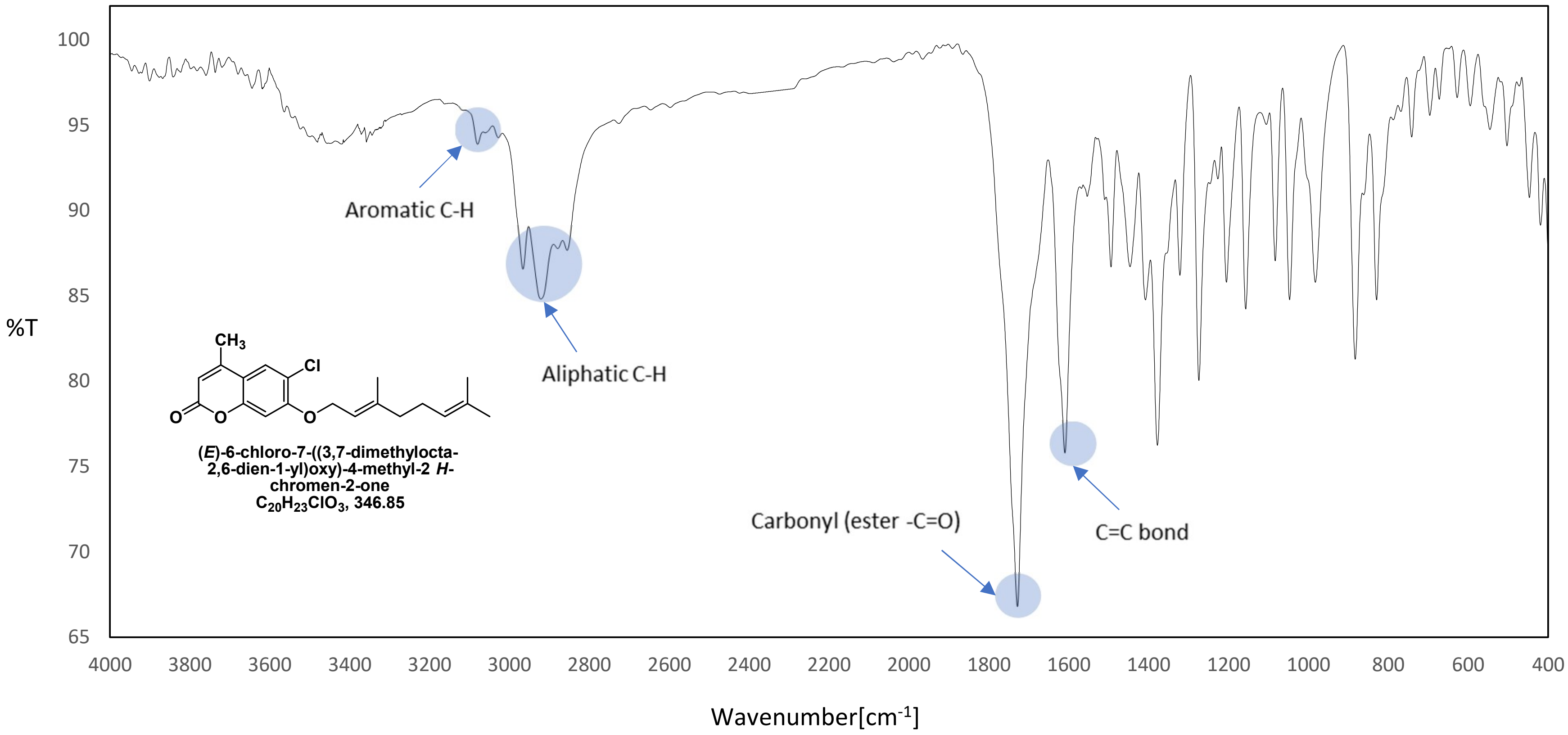

IR(KBr): 3078(Aromatic C-H), 3003 Aromatic C-H), 2965(Aliphatic C-H), 2916(Aliphatic C-H), 2856(Aliphatic C-H), 2854(Aliphatic C-H), 1728(Carbonyl (ester -C=O)), 1609(C=C bond), 1494, 1414, 1388, 1378, 1320, 1274, 1205, 1157, 1083, 1047, 982, 883, 829 cm^{-1}
