# Supplementary figures and images for: Inhibitory Effects of Coumarin Derivatives on Tyrosinase
Source: Molecules. 2021 Apr 17;26(8):2346. doi: 10.3390/molecules26082346 (PMC8073051; doi:10.3390/molecules26082346)

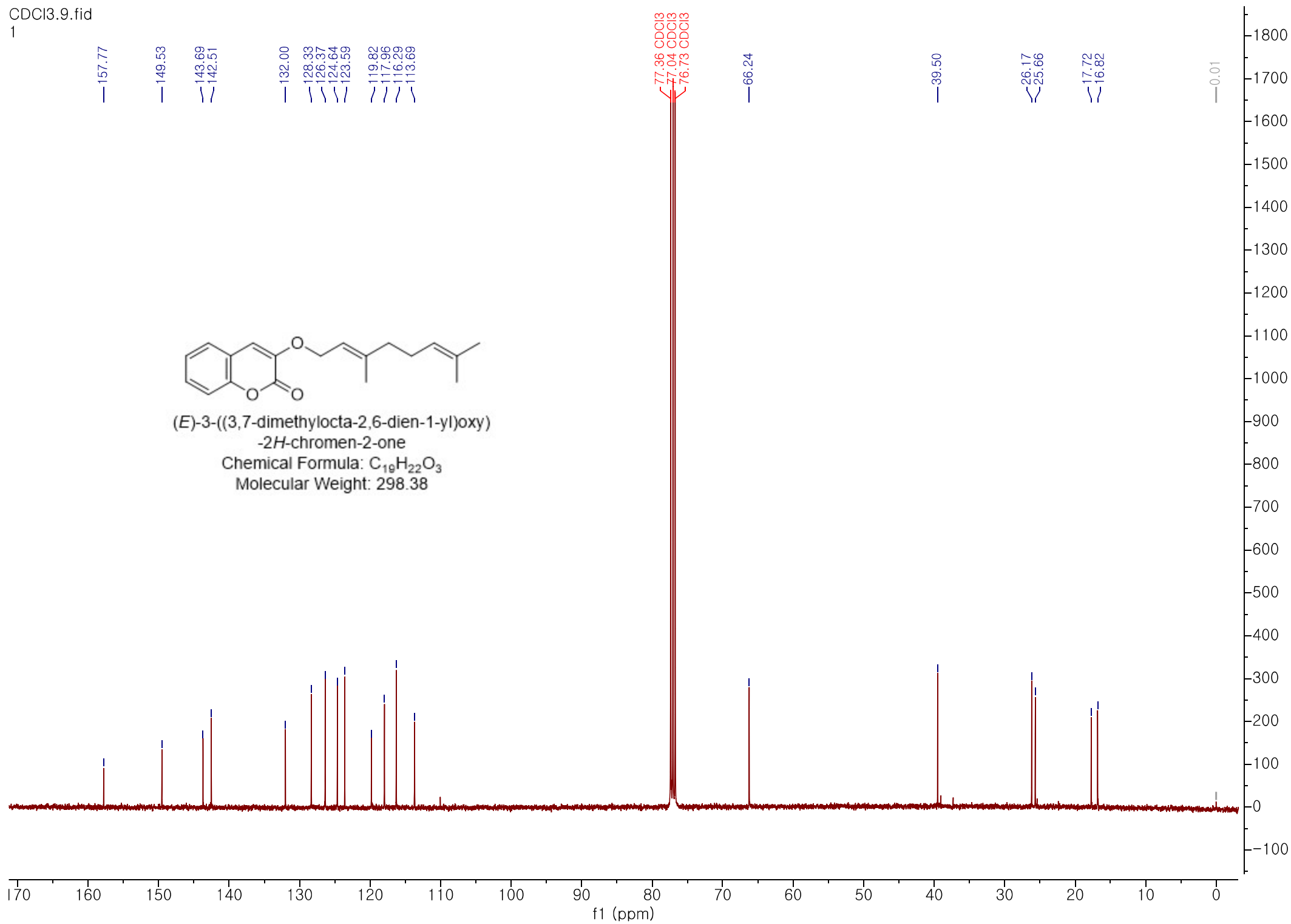

Supplement: Supplementary file 1 [file molecules-26-02346-s001.zip › 3e-C NMR.pdf]

CDCl3.10.fid

1

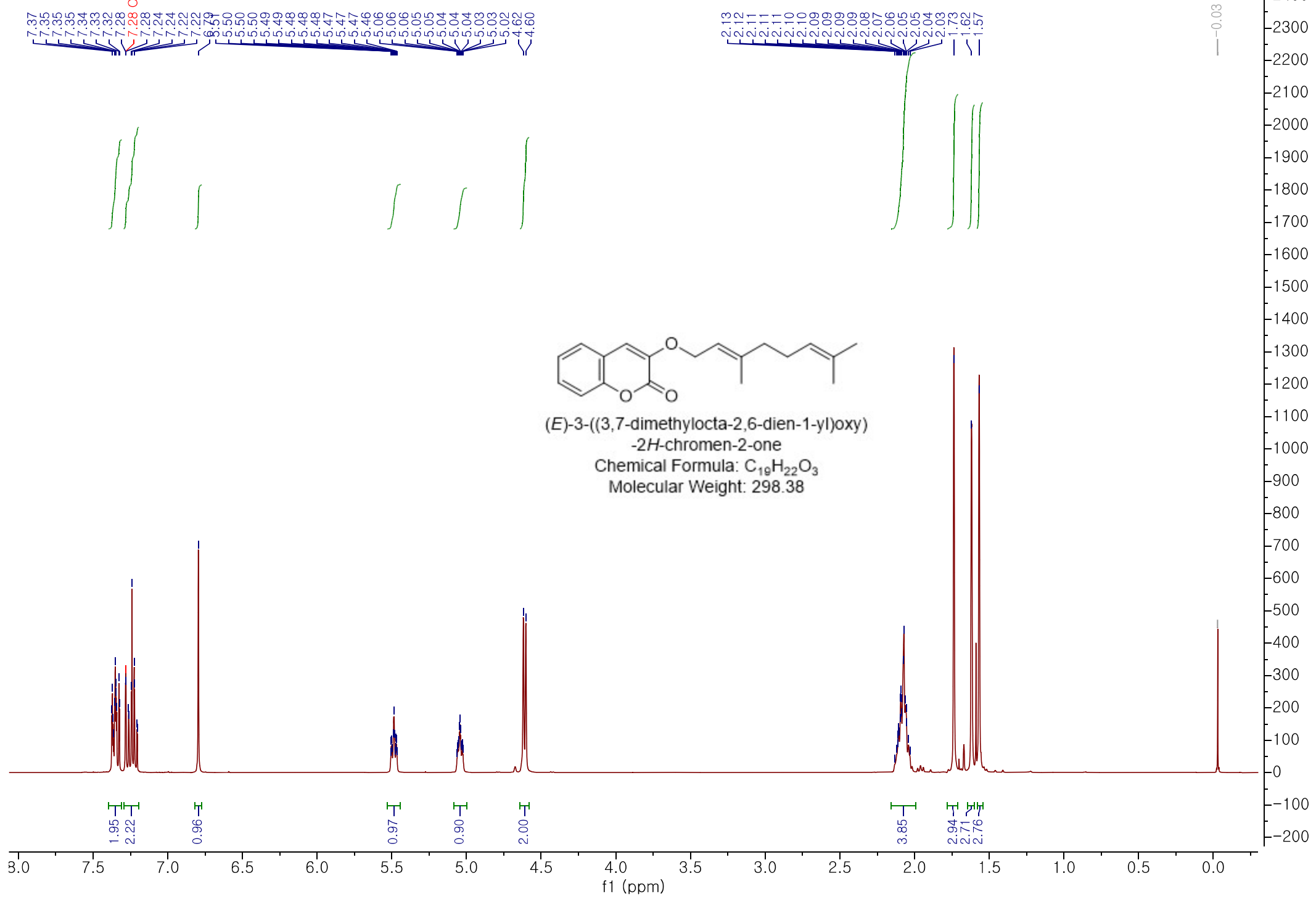

Supplement: Supplementary file 1 [file molecules-26-02346-s001.zip › 3e-H NMR.pdf]

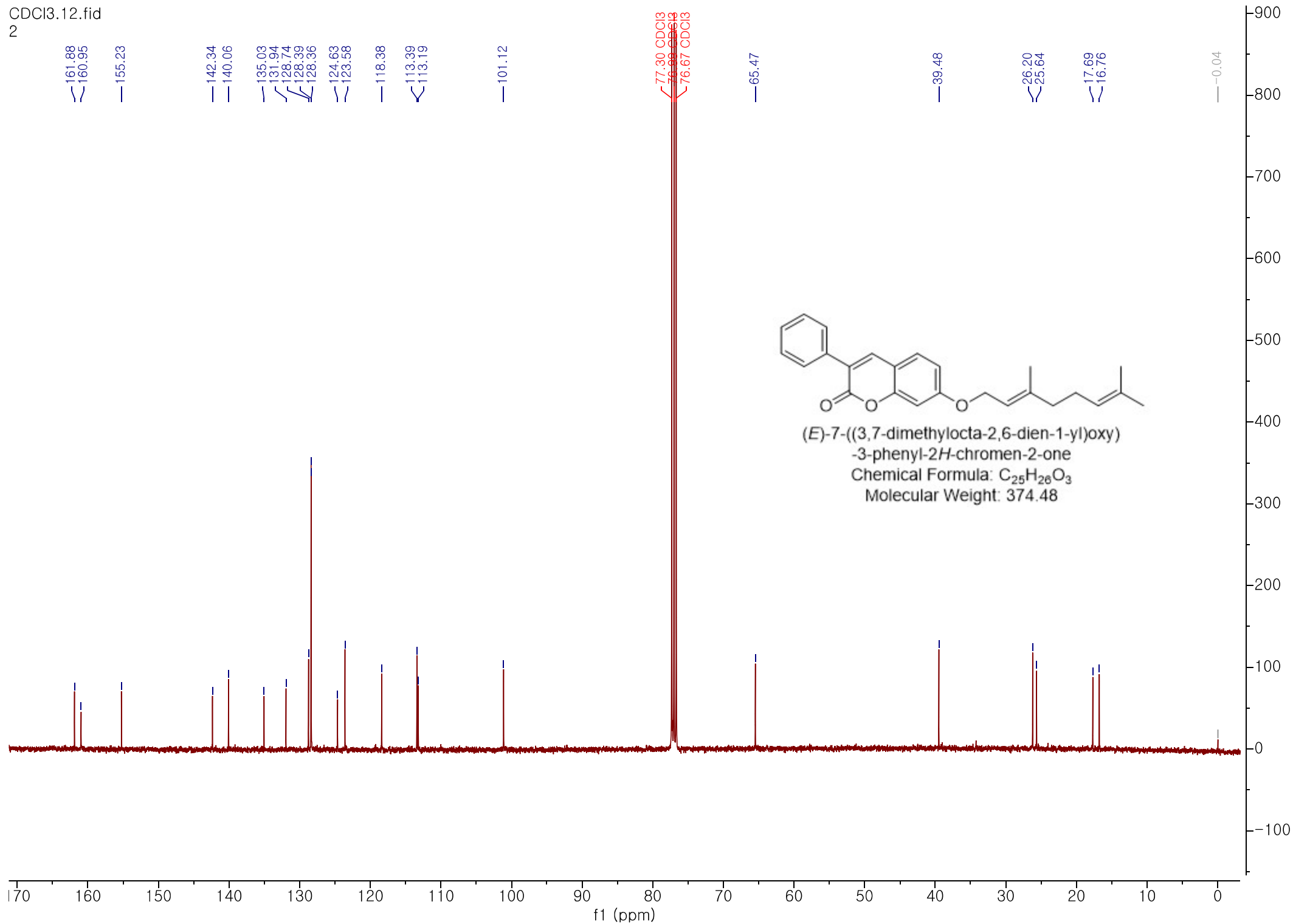

Supplement: Supplementary file 1 [file molecules-26-02346-s001.zip › 3f-C NMR.pdf]

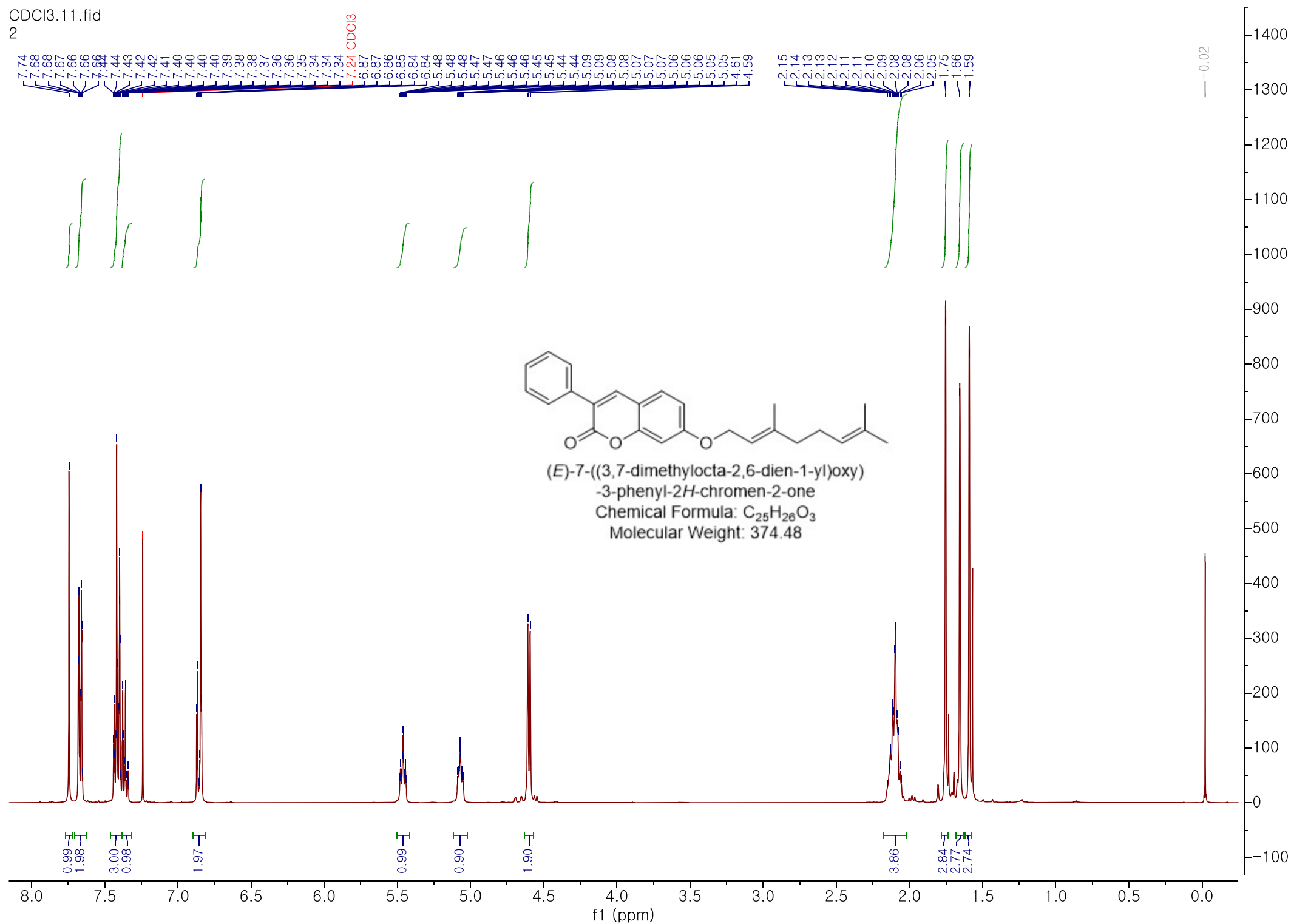

Supplement: Supplementary file 1 [file molecules-26-02346-s001.zip › 3f-H NMR.pdf]

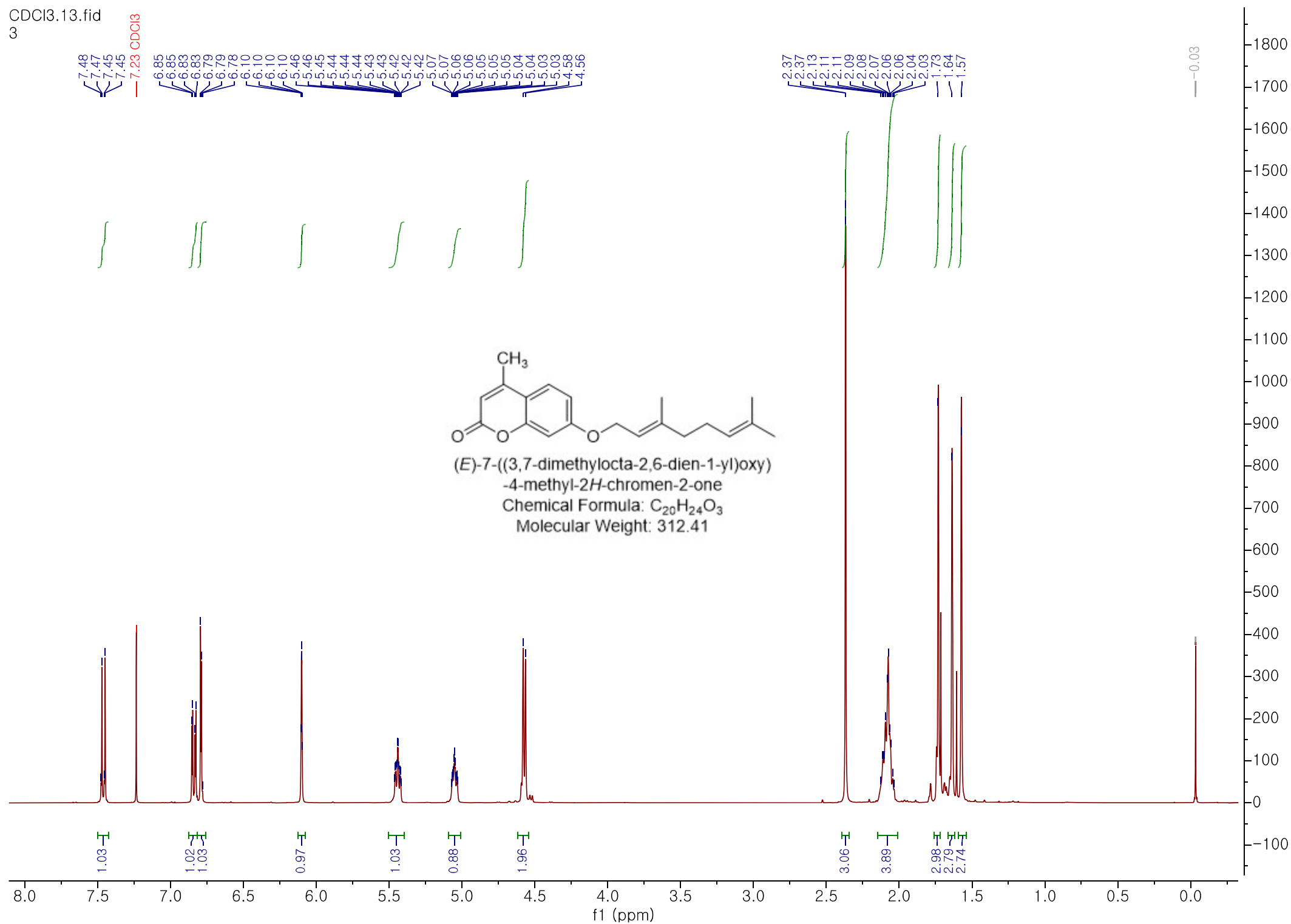

Supplement: Supplementary file 1 [file molecules-26-02346-s001.zip › 3g-H NMR.pdf]

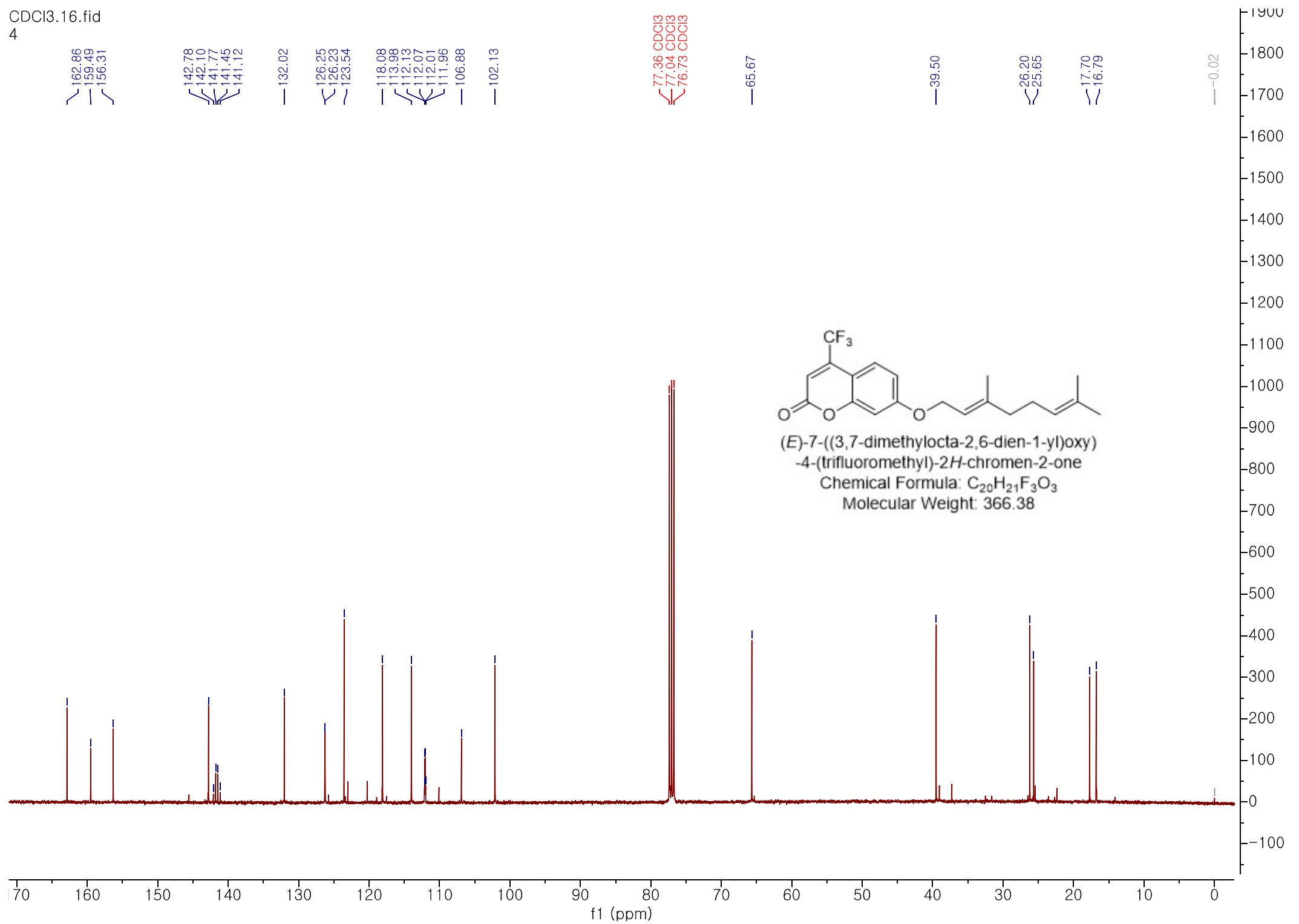

Supplement: Supplementary file 1 [file molecules-26-02346-s001.zip › 3h-C NMR.pdf]

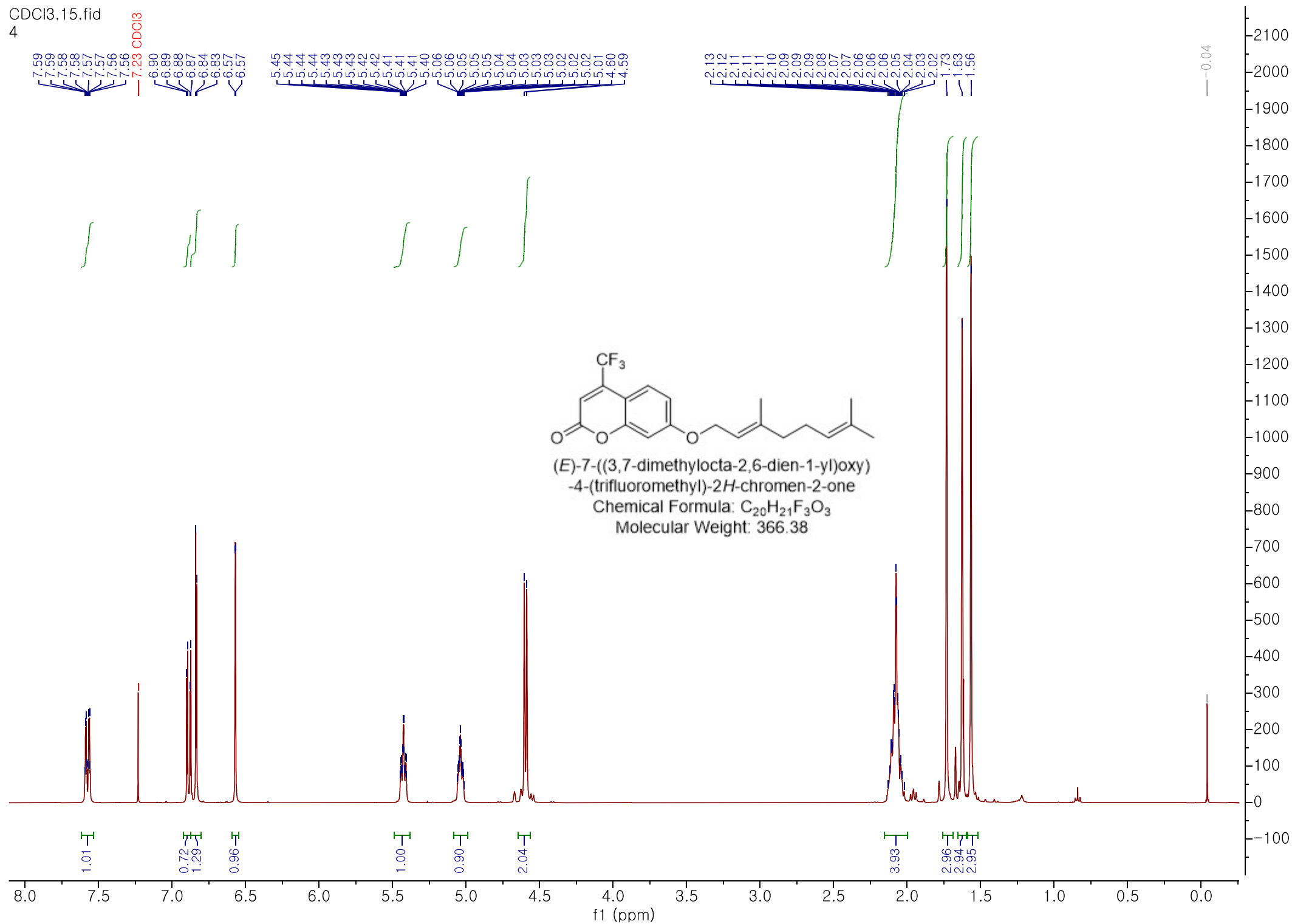

Supplement: Supplementary file 1 [file molecules-26-02346-s001.zip › 3h-H NMR.pdf]

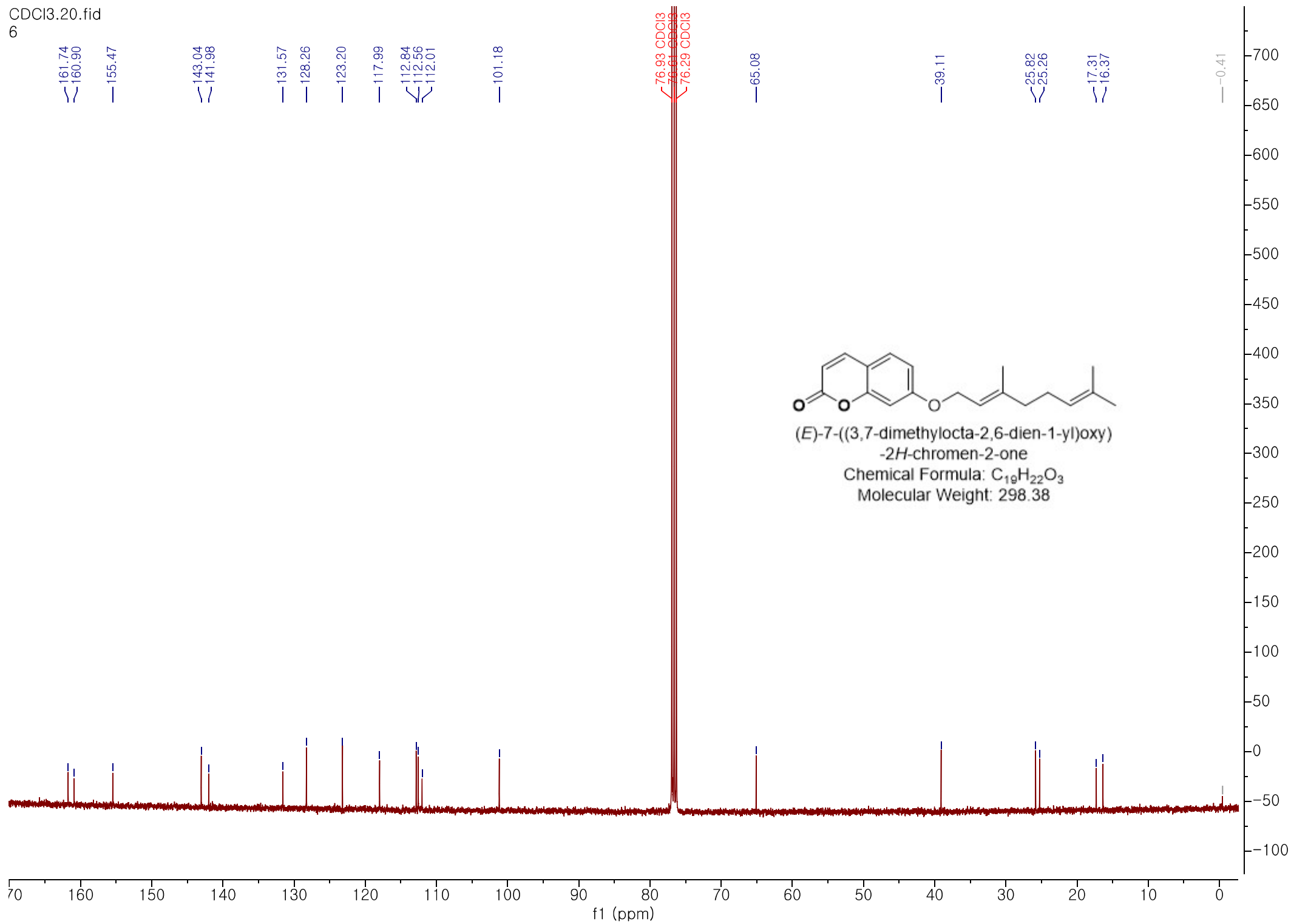

Supplement: Supplementary file 1 [file molecules-26-02346-s001.zip › 3i-C NMR.pdf]

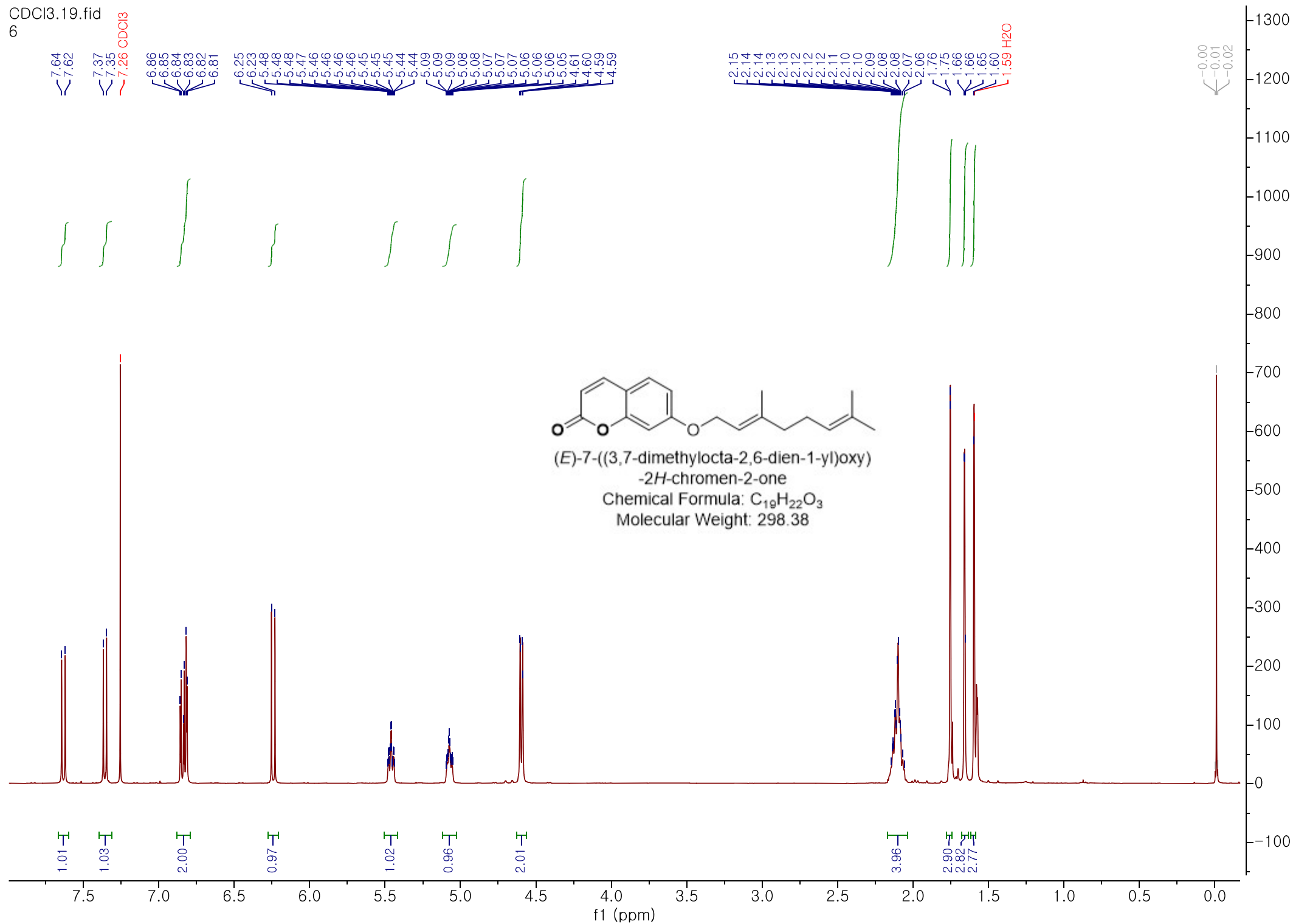

Supplement: Supplementary file 1 [file molecules-26-02346-s001.zip › 3i-H NMR.pdf]

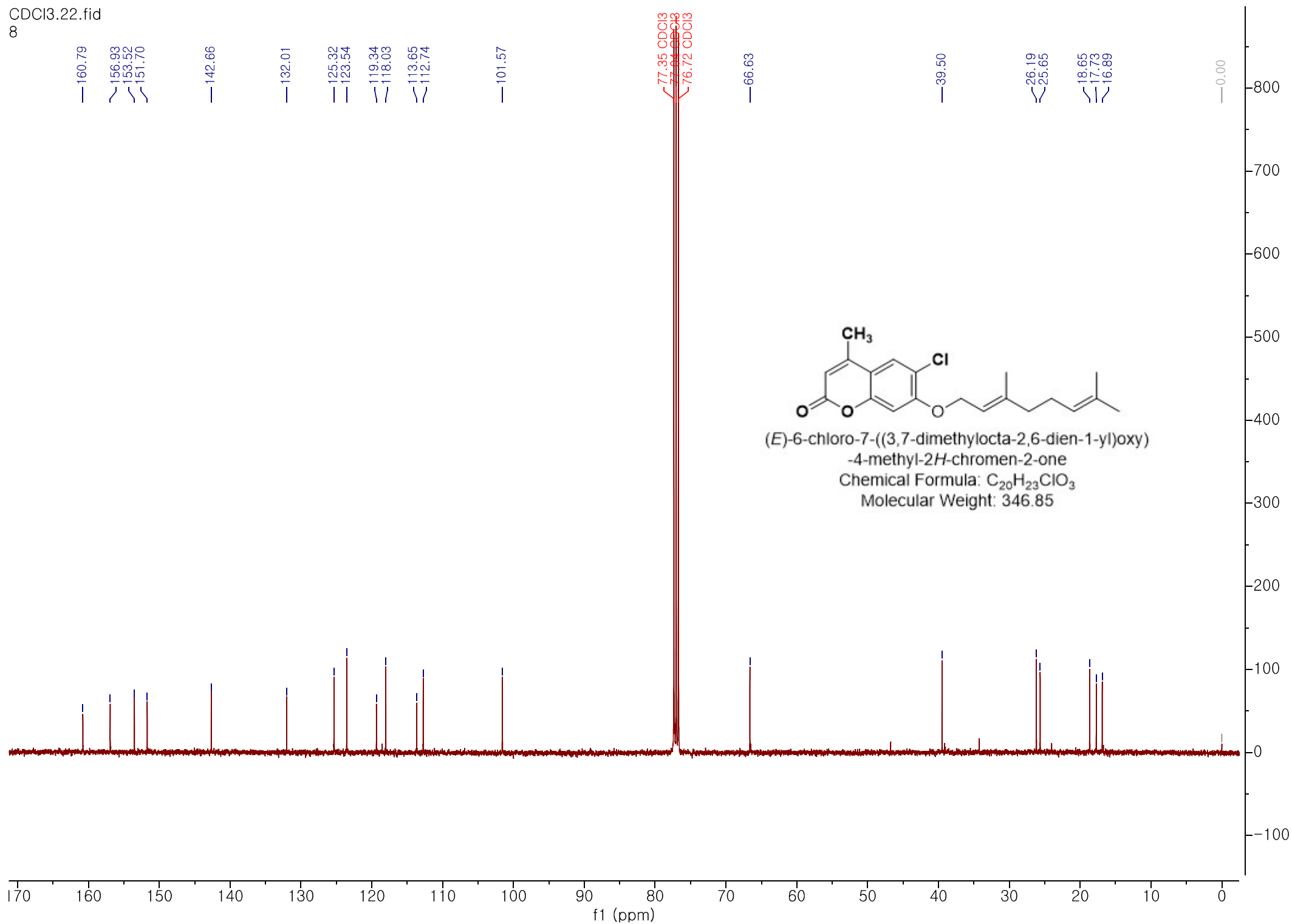

Supplement: Supplementary file 1 [file molecules-26-02346-s001.zip › 3j-C NMR.pdf]

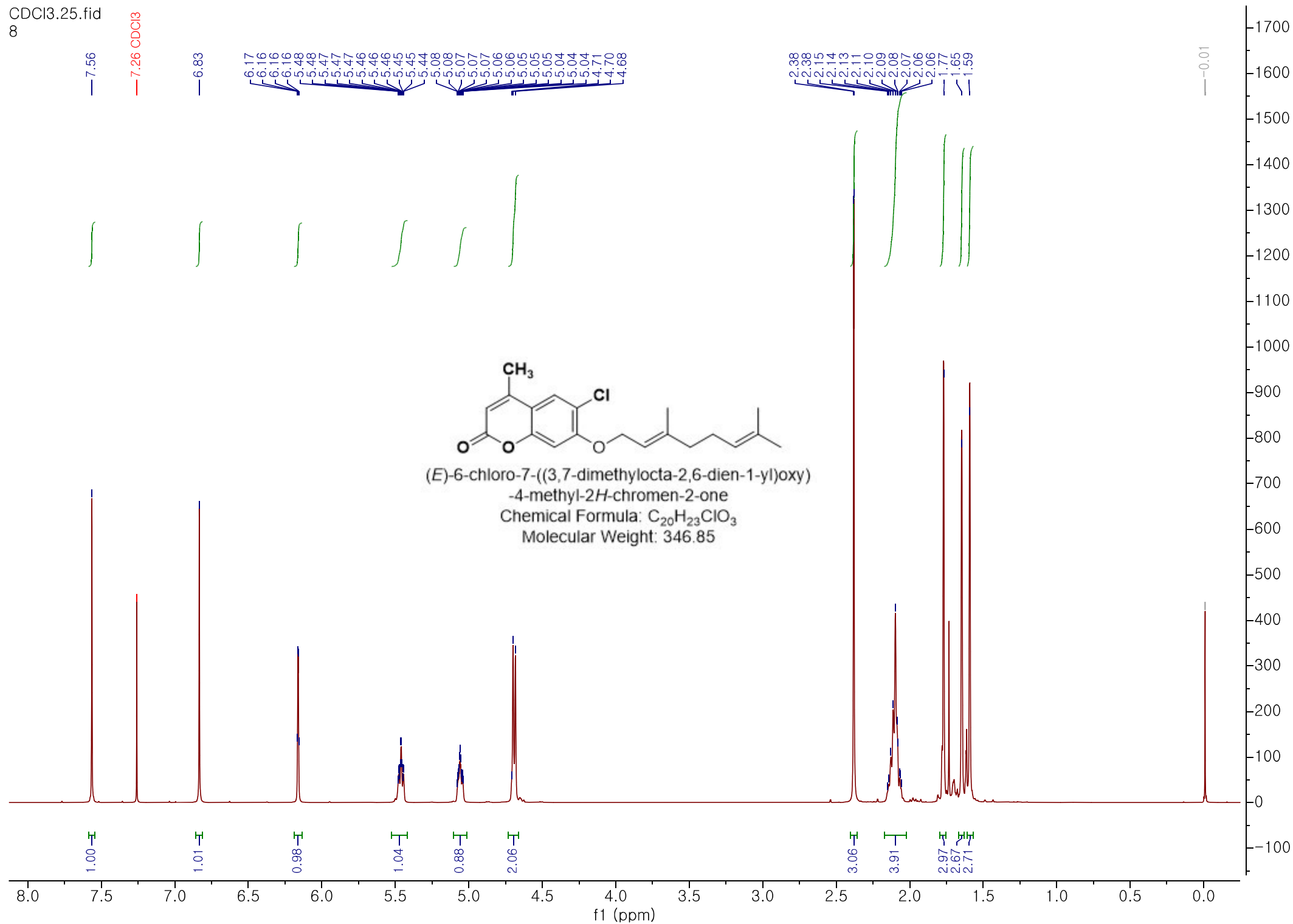

Supplement: Supplementary file 1 [file molecules-26-02346-s001.zip › 3j-H NMR.pdf]

— 7.26 CDCl3

— 6.96

— 6.81

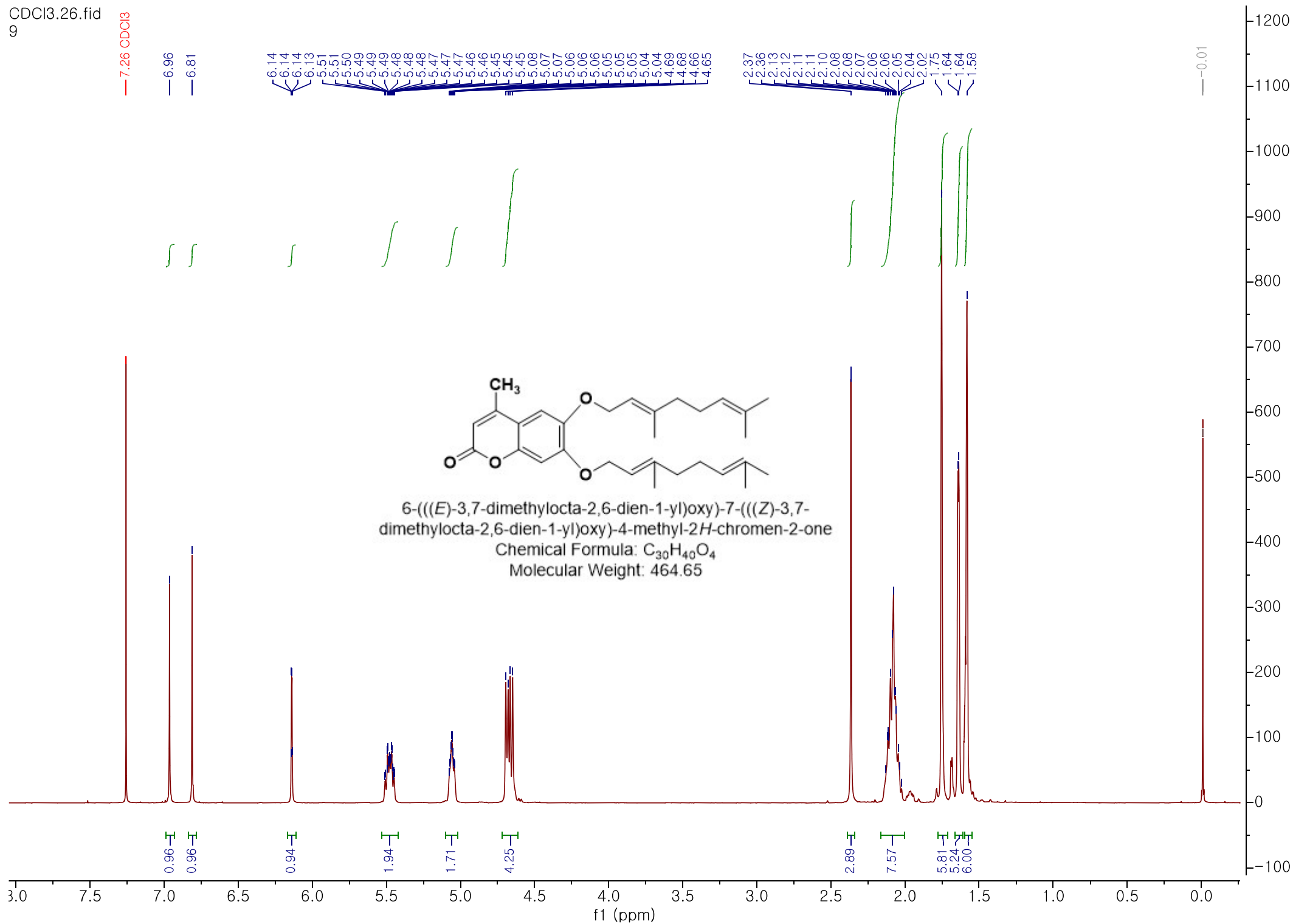

Supplement: Supplementary file 1 [file molecules-26-02346-s001.zip › 3k-H NMR.pdf]

CDCl3.39.fid  
15

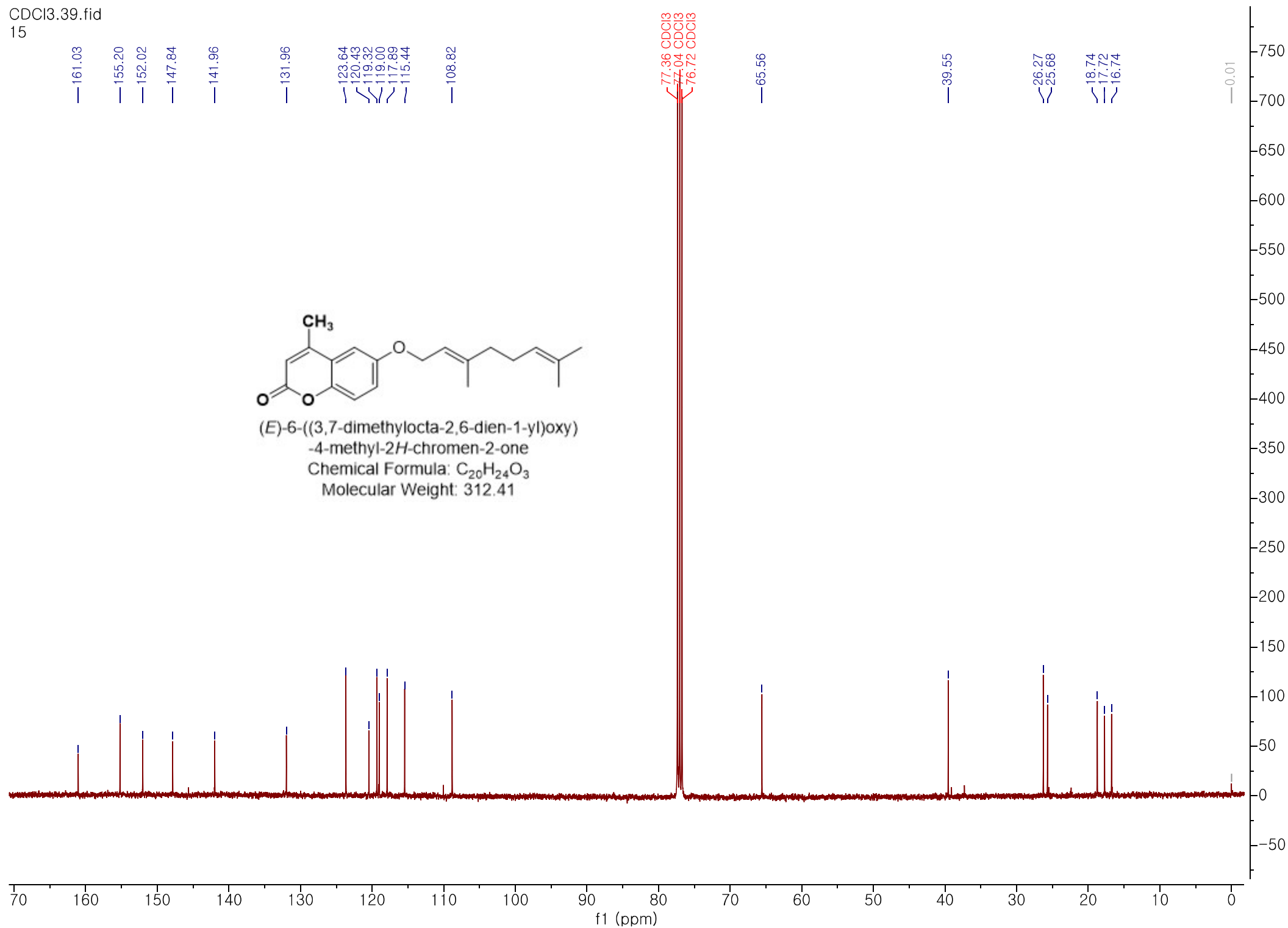

Supplement: Supplementary file 1 [file molecules-26-02346-s001.zip › 3l-C NMR.pdf]

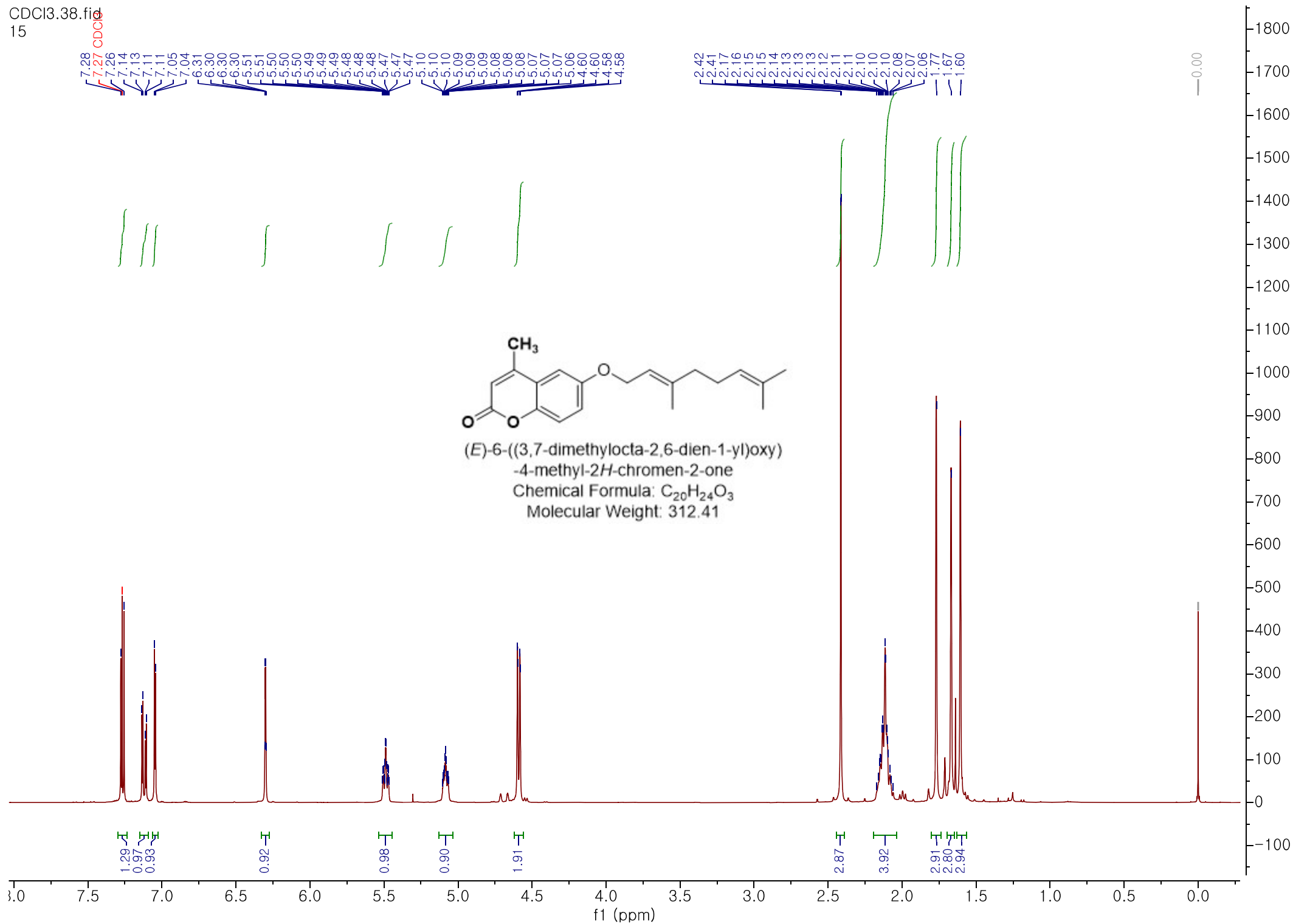

Supplement: Supplementary file 1 [file molecules-26-02346-s001.zip › 3l-H NMR.pdf]

No.3

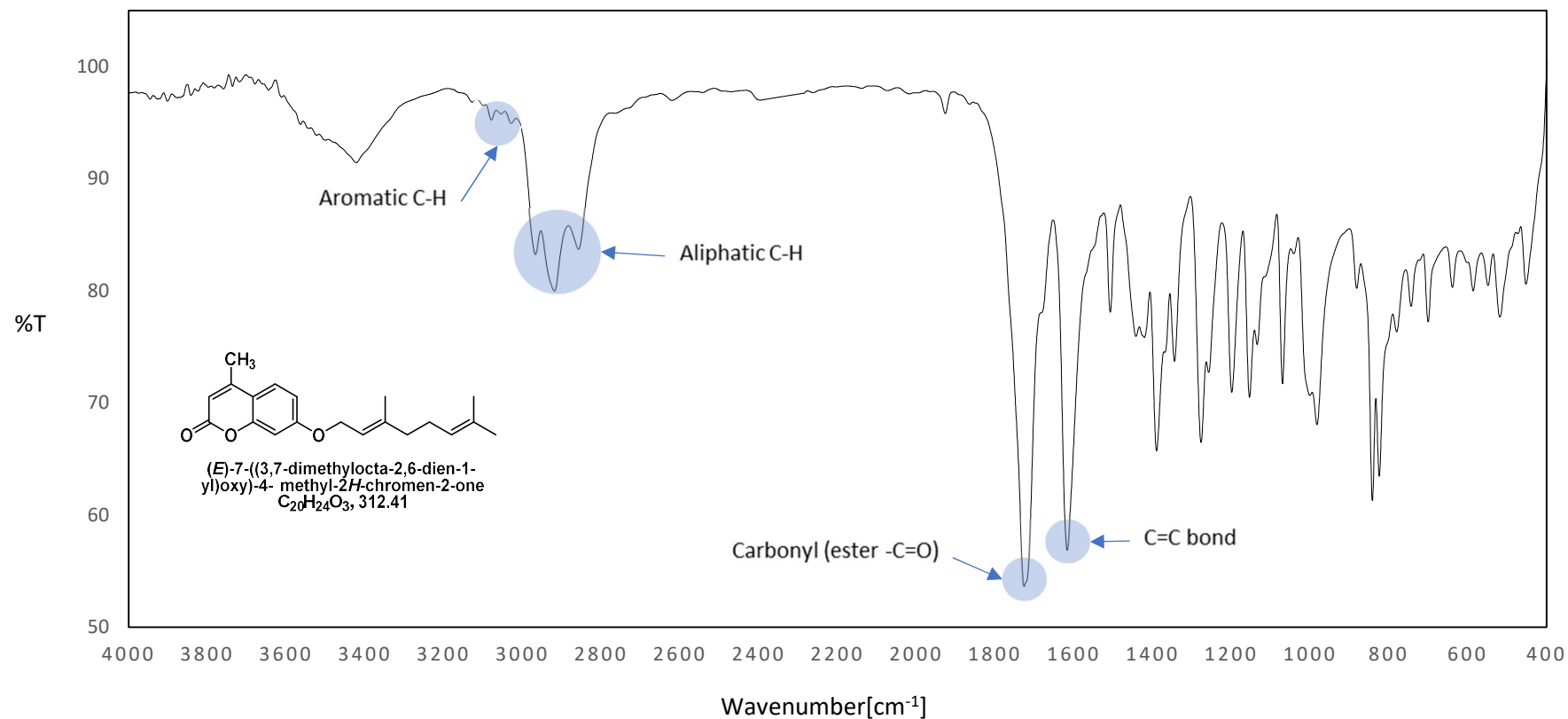

Supplement: Supplementary file 1 [file molecules-26-02346-s001.zip › ir-3g.pdf]

No.9

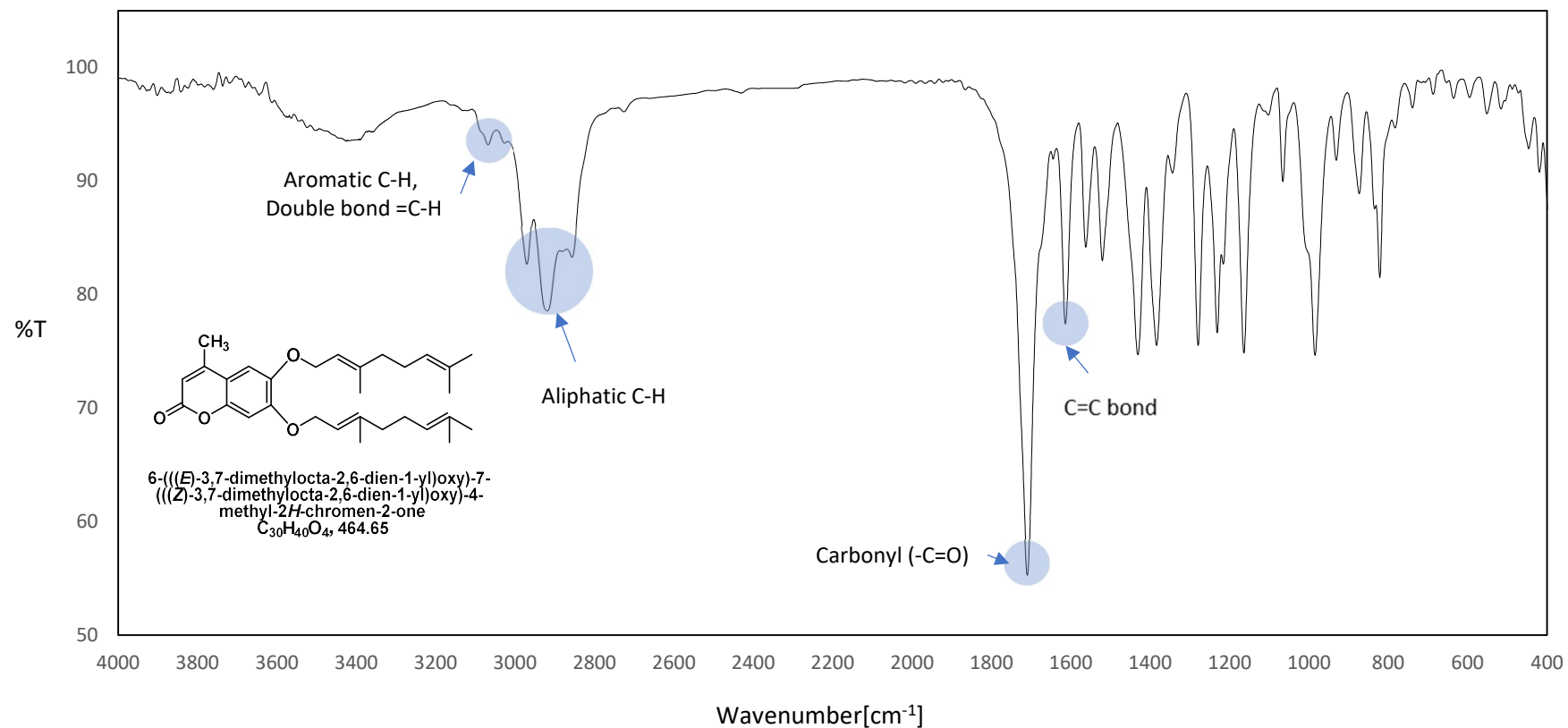

IR(KBr): 3065(Aromatic C-H), 2937(Aliphatic C-H), 2917(Aliphatic C-H), 2854(Aliphatic C-H), 1708(Carbonyl (ester -C=O)), 1612(C=C bond), 1562, 1520, 1430, 1384, 1280, 1231, 1164, 984, 822 cm<sup>-1</sup>

Supplement: Supplementary file 1 [file molecules-26-02346-s001.zip › ir-3k.pdf]

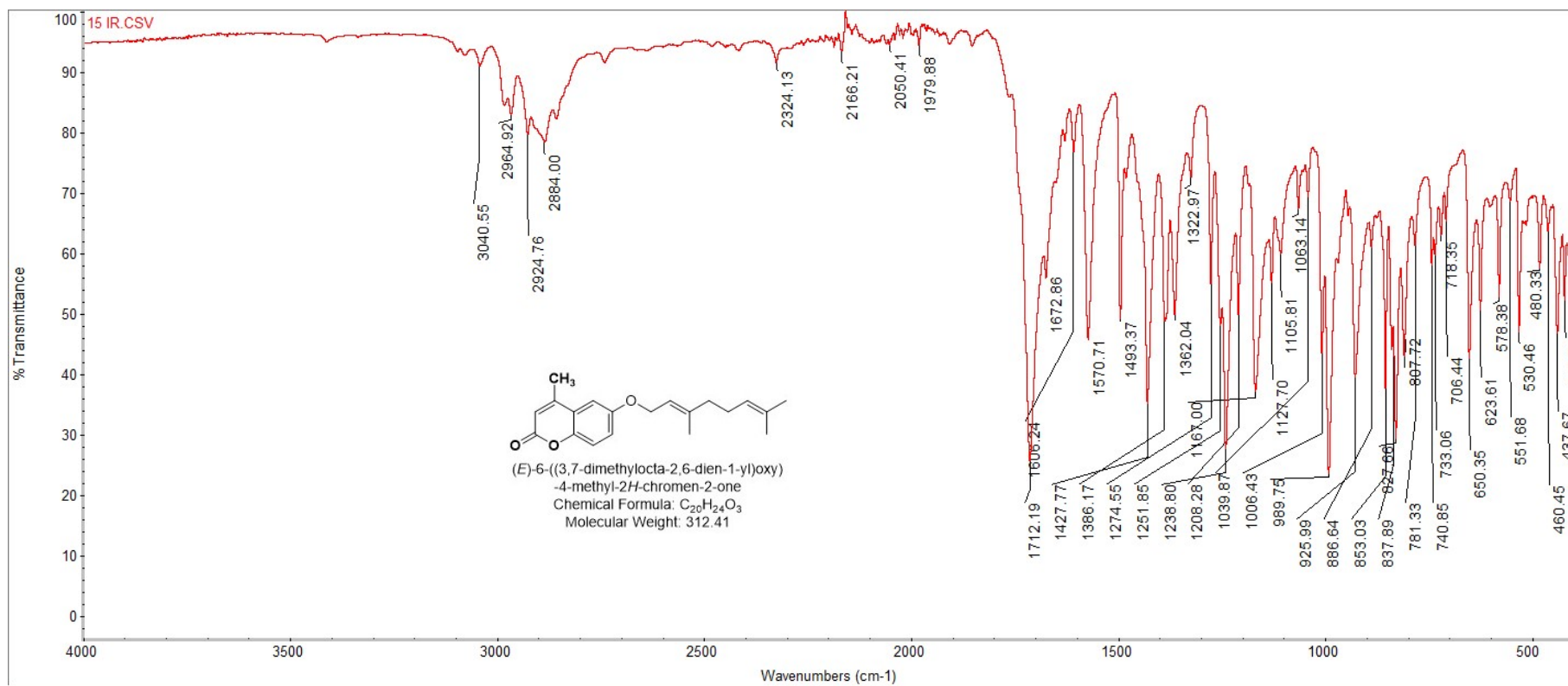

Supplement: Supplementary file 1 [file molecules-26-02346-s001.zip › ir-3l.pdf]
